# Genome-wide discovery of novel M1T1 group A streptococcal determinants important for fitness and virulence during soft-tissue infection

**DOI:** 10.1371/journal.ppat.1006584

**Published:** 2017-08-23

**Authors:** Yoann Le Breton, Ashton T. Belew, Jeffrey A. Freiberg, Ganesh S. Sundar, Emrul Islam, Joshua Lieberman, Mark E. Shirtliff, Hervé Tettelin, Najib M. El-Sayed, Kevin S. McIver

**Affiliations:** 1 Department of Cell Biology & Molecular Genetics and Maryland Pathogen Research Institute, University of Maryland, College Park, Maryland, United States of America; 2 Center for Bioinformatics and Computational Biology, University of Maryland, College Park, Maryland, United States of America; 3 Graduate Program in Life Sciences, University of Maryland School of Medicine, Baltimore, Maryland, United States of America; 4 Division of Infectious Diseases, University of Maryland School of Medicine, Baltimore, Maryland, United States of America; 5 Department of Microbiology and Immunology, University of Maryland School of Medicine, Baltimore, Maryland, United States of America; 6 Department of Microbial Pathogenesis, Dental School, University of Maryland, Baltimore, Maryland, United States of America; 7 Institute for Genome Sciences, University of Maryland School of Medicine, Baltimore, Maryland, United States of America; Boston Children's Hospital, UNITED STATES

## Abstract

The Group A Streptococcus remains a significant human pathogen causing a wide array of disease ranging from self-limiting to life-threatening invasive infections. Epithelium (skin or throat) colonization with progression to the subepithelial tissues is the common step in all GAS infections. Here, we used transposon-sequencing (Tn-seq) to define the GAS 5448 genetic requirements for *in vivo* fitness in subepithelial tissue. A near-saturation transposon library of the M1T1 GAS 5448 strain was injected subcutaneously into mice, producing suppurative inflammation at 24 h that progressed to prominent abscesses with tissue necrosis at 48 h. The library composition was monitored *en masse* by Tn-seq and ratios of mutant abundance comparing the output (12, 24 and 48 h) versus input (T_0_) mutant pools were calculated for each gene. We identified a total of 273 subcutaneous fitness (*scf*) genes with 147 genes (55 of unknown function) critical for the M1T1 GAS 5448 fitness *in vivo*; and 126 genes (53 of unknown function) potentially linked to *in vivo* fitness advantage. Selected *scf* genes were validated in competitive subcutaneous infection with parental 5448. Two uncharacterized genes, *scfA* and *scfB*, encoding putative membrane-associated proteins and conserved among Gram-positive pathogens, were further characterized. Defined *scfAB* mutants in GAS were outcompeted by wild type 5448 *in vivo*, attenuated for lesion formation in the soft tissue infection model and dissemination to the bloodstream. We hypothesize that *scfAB* play an integral role in enhancing adaptation and fitness of GAS during localized skin infection, and potentially in propagation to other deeper host environments.

## Author summary

The WHO ranks the Group A Streptococcus (GAS) in the top 10 leading causes of morbidity and mortality from infectious diseases worldwide. GAS is a strict human pathogen causing both benign superficial infections as well as life-threatening invasive diseases. All GAS infections begin by colonization of an epithelium (throat or skin) followed by propagation into subepithelial tissues. The genetic requirements for M1T1 GAS 5448 within this niche were interrogated by *in vivo* transposon sequencing (Tn-seq), identifying 273 subcutaneous fitness (*scf*) genes with 108 of those previously of “unknown function”. Two yet uncharacterized genes, *scfA* and *scfB*, were shown to be critical during GAS 5448 soft tissue infection and dissemination into the bloodstream. Thus, this study improves the functional annotation of the GAS genome, providing new insights into GAS pathophysiology and enhancing the development of novel GAS therapeutics.

## Introduction

The Group A Streptococcus (*Streptococcus pyogenes*, GAS) is a strict human pathogen of high prevalence worldwide [1–4]. The WHO ranks GAS in the top 10 leading causes of morbidity and mortality from infectious diseases, responsible for over 500,000 deaths annually [5]. Mucosal (throat) and epithelial (skin) surfaces represent GAS primary ecological niches, where GAS causes over 700 million reported cases of purulent, self-limiting infections (*e*.*g*., pharyngitis, impetigo) worldwide each year [4–6]. GAS can also gain access to normally sterile sites of the body (*e*.*g*., soft tissue, bloodstream) and produce life-threatening invasive diseases (necrotizing fasciitis and streptococcal toxic shock syndrome) [7–9]. GAS infections may also trigger the immune sequela acute rheumatic fever, a serious health threat in developing countries [6].

To successfully infect its human host, GAS must adapt to the different niches encountered during the infection process, including physicochemical environmental changes, fluctuating metabolic sources [10–13], as well as the immune response [14]. Molecular epidemiology studies have revealed that GAS pathogenesis is complex [for review, see [15]] with multiple GAS strains (over 230 distinct *emm* types) harboring distinct genetic determinants for tissue tropism and virulence potential [15–17]. Since the first complete genome of the M1 strain SF370 was released in 2001 [18], more than 53 GAS chromosomes have been completed and over 300 draft genomes are available as of June, 2017 (https://www.ncbi.nlm.nih.gov/genome/genomes/175). The GAS genome is genetically diverse with an average size of 1.85 Mb encoding *ca*. 1820 genes [15, 19]. Comparative genomics analyses established that the GAS pan-genome contains over 3900 genes with a set of *ca*. 1200 core genes [15]. A large proportion of GAS genes are still annotated as having no known or predicted function; and accurate functional annotation of GAS genes, particularly in the context of disease manifestations, is key to understanding GAS pathogenesis and improving diagnostics and therapeutics [20].

To investigate pathogenesis of the human-restricted GAS, *in vivo* infection models have been developed mostly using mice as the host and specific virulent GAS strains capable of producing human-like disease symptoms in these murine models [4, 21–26]. Historically, GAS isolates from the globally distributed M1T1 serotype have been commonly used to investigate GAS virulence potential *in vivo*. Among these, GAS strain 5448 is a clinical isolate representative of the M1T1 serotype that has been successfully employed in different mouse models of skin and tissue infections as well as necrotizing fasciitis [27–34]. GAS 5448 is also a model organism to study the increased virulence potential caused by mutations in the *covS* gene, *i*.*e*. the *covS* switch, during invasive infections by the M1T1 serotype [27, 34–36]. Furthermore, GAS 5448 genome sequence was recently released [37]. However, as found with many other GAS strains that are relevant to study virulence in *in vivo* mouse infection models, genetic manipulations of 5448 can prove difficult [38].

To accelerate functional genomics analyses of GAS pathogenesis, we developed a *mariner* transposon system (*Oskar*) for GAS to perform highly saturated mutagenesis [38] and subsequent high-throughput phenotype screens. Initially, we employed a transposon site hybridization (TraSH) screen to identify genes required for GAS 5448 fitness in an *ex vivo* human blood infection model [39–41]. To take advantage of increased sensitivity and resolution, we modified our *mariner* (*Krmit*) for transposon-sequencing (Tn-seq) [42], a method that uses massive parallel DNA sequencing to assay the frequency of transposon insertion sites within complex mutant libraries. Using Tn-seq, we were able to create highly saturated *Krmit* libraries in both M1T1 5448 and M49 NZ131 serotypes and define the GAS minimal core genome (essential, *i*.*e*., non-mutable, genes) for *in vitro* growth in rich media [43].

In this report, we present a genome-wide Tn-seq analysis to identify M1T1 GAS 5448 genetic determinants necessary for *in vivo* fitness using a murine model of skin and soft tissue infection. Immunocompetent hairless mice were subcutaneously inoculated with an M1T1 GAS 5448 *Krmit* library, and gene fitness was assessed from infected tissues to identify mutants differentially represented between the input (T_0_) and various output pools (12, 24 and 48 HPI) representing clinical progression from inflamed focus of infection to tissue destructive abscess. A total of 273 subcutaneous fitness (*scf*) genes related to M1T1 GAS 5448 fitness were identified (147 and 126 genes associated with decreased and increased fitness, respectively) in the subcutaneous environment, with 108 of those genes annotated as “of unknown function”. To validate our Tn-seq dataset, defined mutants were created in selected *scf* genes and tested using *in vivo* competition with parental GAS 5448 in the soft tissue of mice. Two such genes, *scfA* and *scfB*, encode putative membrane-associated proteins that are conserved among Gram-positive pathogens and were important for GAS fitness in the lesion at both 24 and 48 HPI. Defined mutants in *scfAB* were outcompeted by wild type GAS 5448 *in vivo*, and were attenuated for lesion formation in the soft tissue infection model as well as for subsequent dissemination into the bloodstream. Thus, ScfAB likely play an integral role in enhancing adaptation and fitness of GAS 5448 during localized skin infection, and potentially in other host environments. Our comprehensive Tn-seq-based dataset of *scf* genes in GAS will allow researchers to define critical pathways and assign functional attributes to the large number of GAS genes annotated as unknown function or hypothetical.

## Results

### Genetic determinants involved in GAS 5448 fitness *in vitro*

**Fig 1:**
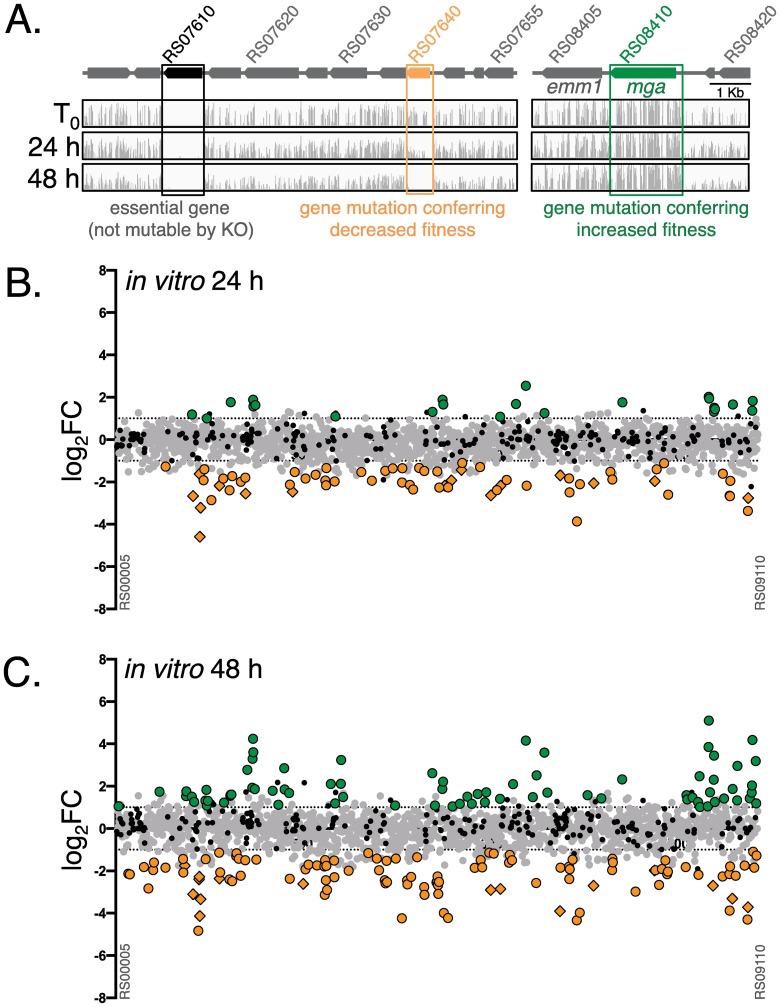
Tn-seq analysis of M1T1 GAS 5448 fitness during *in vitro* growth in THY. (A) Schematic of two representative chromosomal regions of M1T1 GAS 5448 (above) with the contribution (below) of GAS genes (arrows) during growth in THY determined by Tn-seq illustrated (IGV) with location (horizontal axis) and depth (vertical axis) of all *Krmit* transposon insertion sites identified in the GAS 5448 *Krmit* library initially (T_0_) and after 24-h and 48-h passages, respectively. Highlighted (boxed) are genes found to have a neutral effect (grey), decreased (orange), or increased (green) survival in the screen. Genes previously identified as essential [43] are shown in black and were not included in the fitness analyses. (B and C) Genome-scale summary of the ratios of mutant abundance (log_2_FC, Y axis) calculated using DEseq2 for each GAS 5448 gene (X axis), comparing the 24 h (B) and 48 h (C) output pools to the T_0_ input mutant pool. Gene mutations conferring decreased (log_2_FC < -1, *p* < 0.05) or increased fitness (log_2_FC > 1, *p* < 0.05) are indicated with orange and green circles, respectively. Neutral mutations (grey circles) and essential genes (black circles) are also indicated.

We recently constructed a near-saturation *mariner* transposon (*Krmit*) mutant library representing *ca*. 85,000 independent insertions in the M1T1 GAS strain 5448 genome that allowed us to combine Tn-seq with a Bayesian statistical model to predict the genes essential for *in vitro* growth in THY rich media [43]. Mutants lost from the input pool only after multiple passages in THY (termed critical) were proposed to represent genes important for fitness *in vitro* [43]. As these genes could alter the interpretation of the proposed *in vivo* Tn-seq screen, we re-analyzed our original Tn-seq datasets [43] obtained from two successive overnight passages (24 and 48 h) of the M1T1 5448 *Krmit* library in THY. Reads were summed for each GAS gene, and the ratios of mutant abundance comparing the output versus input mutant pools (fold change, FC) for each gene was calculated using the DEseq2 pipeline (see Material and methods). Results for 24 h and 48 h passages compared to the input (T_0_) were expressed using a log base 2 transformation of the FC (log_2_FC in S1 Table). Essential genes identified previously [43] (Fig 1, black) were removed from the current analyses. Genes with fewer insertions in the output pool (log_2_FC < -1, *p* < 0.05) were considered to confer decreased fitness (Fig 1, orange); those showing increased numbers of mutants in the output pools (log_2_FC > 1, *p* < 0.05) conferred a selective advantage or increased fitness (Fig 1, green), while genes with no significant change were considered neutral (Fig 1, grey).

Analysis of M1T1 GAS 5448 *in vitro* fitness in THY identified 65 (24 h) and 112 (48 h) genes associated with decreased *in vitro* fitness, with 38 genes common to both time points (Fig 1B and 1C, orange circles; S1A Fig). Conversely, 23 (24 h) and 75 (48 h) genes were linked to increased *in vitro* fitness in THY, with 21 genes common to both time points (Fig 1B and 1C, green circles; S1B Fig). Clusters of Orthologous Genes (COG) enrichment analyses of genes identified during our *in vitro* Tn-seq analysis (n = 216) revealed that 65 (30%) encoded for proteins of unknown function, the highest COG for both increased and decreased fitness (S1C Fig). Additional genes linked to decreased *in vitro* fitness were primarily involved in the transport and metabolism of amino acids (~10%), inorganic ions (~8%), carbohydrates (~6%), and nucleotides (~6%) (S1C Fig). For increased *in vitro* fitness, gene products were also mostly related to the transport and metabolism of nucleotides (~10%), amino acids (~5%), and carbohydrates (~5%), as well as DNA replication (~8%) and signal transduction (~6%) (S1C Fig).

### Validation of the *in vitro* Tn-seq screen

**Fig 2:**
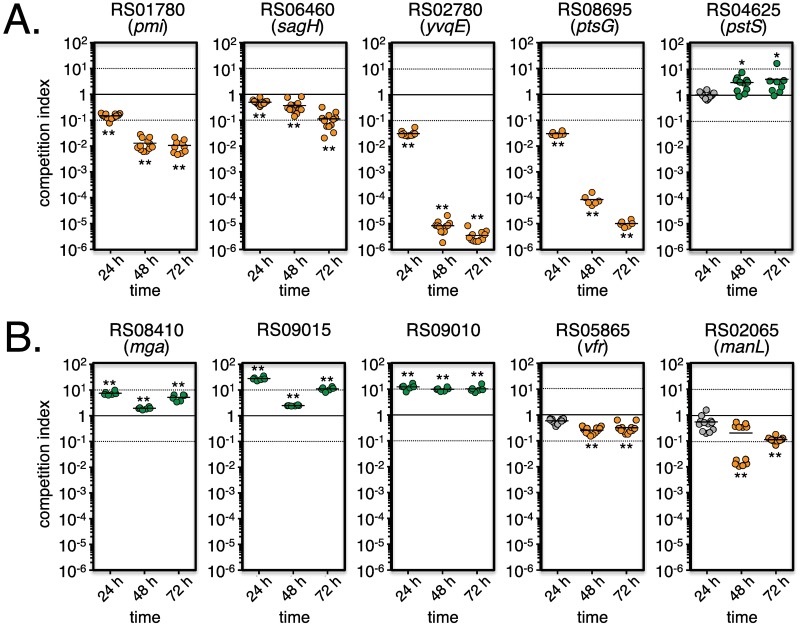
Validation of the *in vitro* Tn-seq screen. Defined mutants (see Material and methods) in selected genes identified by Tn-seq as linked to decreased (A) or increased (B) fitness *in vitro* were grown in THY broth with GAS 5448 (*ca*. 1:1 ratio) in competitive growth assays. CFU counts were determined and strain ratios monitored after three successive 24-h passages (24 h, 48 h, 72 h) and expressed as competitive indexes. Results that validate the *in vitro* Tn-seq data are indicated as decreased (orange circles), increased (green circles), and neutral (grey circles) fitness in competitive growth assays. Unpaired student’s *t*-test was used to evaluate the significance of differences between groups; a *p* value of <0.05 (*) or <0.01 (**) was considered statistically significant.

To provide experimental validation for our dataset, we selected 5 genes associated with decreased GAS fitness (RS01780/*pmi*, RS06455/*sagH*, RS02780/*yvqE*, RS08695/*ptsG* and RS04625/*pstS*) and 5 genes linked to increased GAS fitness (RS08410/*mga*, RS09015, RS09010, RS05865/*vfr* and RS02065/*manL*). Defined mutants were produced by insertional inactivation (see Material and methods) and growth assays using pure cultures in THY revealed growth parameters comparable to wild type (WT) 5448. To mimic the *en masse* Tn-seq screen, competition growth assays were performed in THY using similar CFU of WT and each mutant (see Material and methods). Population composition was monitored after plating serial dilutions after 3 successive overnight passages (*i*.*e*., 24, 48 and 72 h). Four of the five tested genes representing decreased *in vitro* fitness (*pmi*, *sagH*, *yvqE*, *ptsG*; Fig 2A) recapitulated the Tn-seq findings whereas *pstS* did not. For the genes showing increased fitness in Tn-seq, three (*mga*, RS09015 and RS09010) out of the 5 genes also demonstrated a positive competition index in direct competition with wild type 5448 *in vitro* (Fig 2B). Attempts to validate the remaining two genes (*vfr* and *manL*) were unsuccessful (Fig 2B). It is possible that the insertional mutagenesis strategy (pSinS, see Material and methods) employed for the *pstS*, *vfr* and *manL* genes could result in a partially functional truncated allele or have a polar effect on downstream genes. Overall, we were able to experimentally validate 70% of the tested genes, providing confidence in our fitness pipeline.

### An *in vivo* model of GAS skin and soft tissue infection suitable for Tn-seq

**Fig 3:**
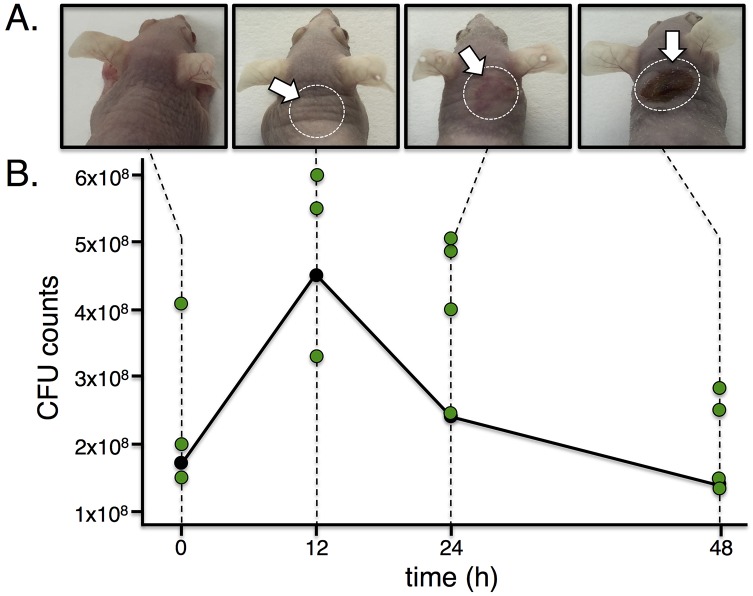
The murine soft tissue infection model and GAS M1T1 5448 are suitable for *in vivo* Tn-seq. (A) Immunocompetent hairless Crl:SKH1-hrBR mice were infected subcutaneously with *ca*. 1–4 x 10^8^ CFU of either wild-type GAS 5448 (shown) or the GAS 5448 *Krmit* library. Both produced marked lesions at 24 h that became necrotic and ulcerative by 48 h (white arrows, dotted circles). (B) Bacterial load within the excised lesion tissue was monitored by total CFU counts over time. The average counts for WT 5448 are displayed as black circles, and individual counts for the *Krmit* libraries in each lesion are indicated with green circles.

To interrogate GAS pathogenesis by Tn-seq, we selected a murine model of GAS subcutaneous infection using outbred immunocompetent hairless Crl:SKH1-hrBR mice [44–46]. In pilot experiments, mice were infected at the base of the neck with *ca*. 2×10^8^ CFU of WT 5448 and progression of the disease followed over 48 h. Visual inspection of the infected tissues revealed the formation of a visible lesion 24 h post infection (HPI) that developed into a necrotic abscess by 48 HPI (Fig 3A). Histopathology of tissue samples from non-infected mice (S2A Fig) did not show any pathological alteration. At 12 HPI, inflammatory infiltrates (*e*.*g*., macrophages) were visible in the dermis, panniculus carnosus muscle (PCM) and hypodermis (S2B Fig, black arrows), with signs of suppurative inflammation associated with early necrosis (S2C Fig). However, the epidermis appeared asymptomatic and GAS cells were not visible (S2B Fig). At 24 HPI, epidermal thickening was observed, even though it was uninvolved in the infection process (S2D Fig). Ongoing inflammation of the hypodermis and necrotic PCM revealed prominent infiltration of macrophages (some intracellular GAS), lymphocytes and neutrophils (S2F Fig, blue, orange and green arrows, respectively); with bacterial growth in devitalized, edematous tissue (S2F Fig, black arrows). At 48 HPI, abscess formation was observed with extensive inflammation and tissue damage forming a pseudocapsule surrounding necrotic debris found in the hypodermis (S2E Fig). GAS chains were visible in the midst of necrotic tissues (S2G Fig, black arrows). Altogether, histopathology revealed that 5448 affected tissues in the deep reticular dermis, PCM and hypodermis, with inflammation visible at 12 and 24 HPI, leading to an abscess formation by 48 HPI in the mouse model of GAS skin and soft tissue infection as previously observed with other GAS strains [44, 45].

To verify that an adequate number of GAS cells could be retrieved from the M1T1 GAS 5448-infected tissues for Tn-seq, the tissue surrounding the site of infection was surgically extracted, homogenized by mechanical disruption and tissue lysates plated on blood agar plates for CFU counts. GAS cell numbers were recoverable in the range of 10^8^ CFUs at the different time points tested (Fig 3B, black circles), a bacterial load that provides the complexity and numbers required for Tn-seq. We also observed that a proportion (15% on average) of the retrieved GAS cells from tissue lysates collected at 48 HPI produced mucoid colonies, a phenotype associated with GAS cells overproducing capsule. This is consistent with published reports showing that M1T1 GAS 5448 can acquire mutations *in vivo* functionally inactivating the *covS* gene (*covS* switch), leading to the derepression of the capsule-encoding *hasABC* operon [34, 36, 47–49].

### Tn-seq analyses of M1T1 GAS 5448 during skin and soft tissue infection

Hairless Crl:SKH1-hrBR mice were infected with the M1T1 GAS 5448 *Krmit* mutant library [43], leading to lesions and tissue damage similar to those produced with WT 5448 (Fig 3, S2 Fig). Retrieval of M1T1 GAS 5448 *Krmit* mutants from excised lesions at 12, 24 and 48 HPI, yielded CFU counts in the 10^8^ range (Fig 3B, green circles) comparable to those observed for wild type 5448 in the pilot experiments. Accumulation of mucoid colonies was also observed at the 24 and 48 HPI lesions, 5% and 25%, respectively. Analysis of selected mucoid colonies by AP-PCR to precisely identify transposon insertion sites revealed multiple independent *Krmit* mutations in the *covS* gene (S3 Fig) that were present in the input library prior to infection.

**Fig 4:**
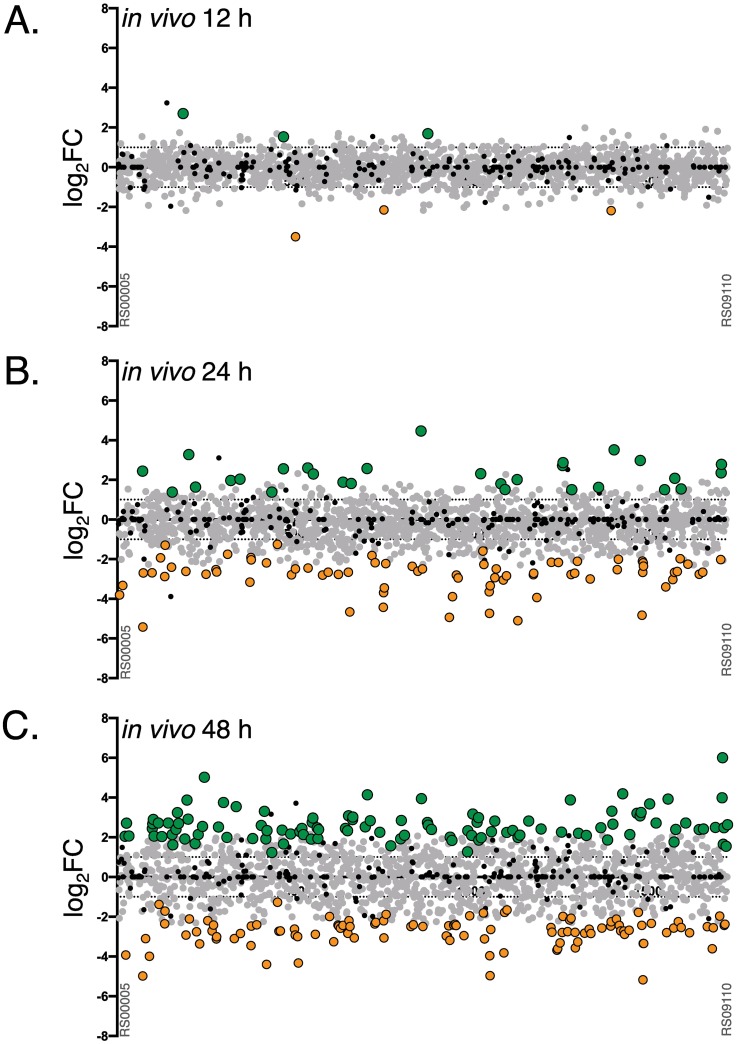
Tn-seq analyses of M1T1 GAS 5448 fitness during murine skin and soft tissue infection. Genome-scale summary of the ratios of mutant abundance (log_2_FC, Y axis) calculated using DEseq2 for each gene (X axis) comparing the GAS 5448 *Krmit* library composition retrieved from *in vivo* lesions after 12, 24 and 48 HPI versus the T_0_ input pools (A, B and C, respectively). Gene mutations conferring decreased (log_2_FC < -1, *p* < 0.05) or increased fitness (log_2_FC > 1, *p* < 0.05) are indicated with orange and green circles, respectively. Neutral mutations (grey circles) and essential genes (black circles) are also indicated.

**Fig 5:**
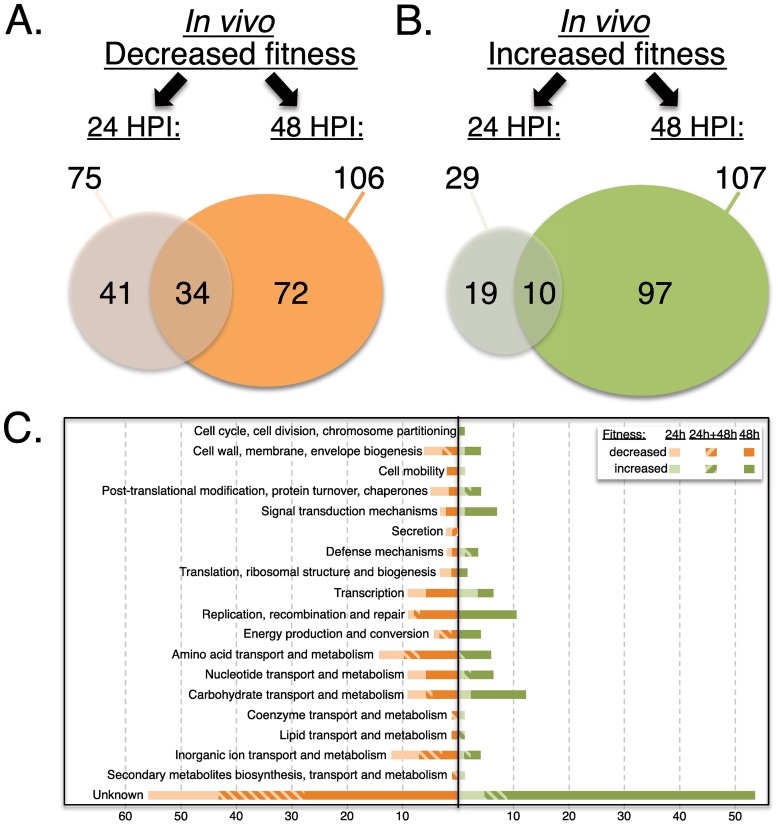
Comparison and classification of genes impacting M1T1 GAS 5448 fitness during *in vivo* lesion formation. Venn diagrams comparing the number of genes showing either decreased (orange) or increased (green) fitness from the Tn-seq analyses of lesions 24 (A) and 48 (B) HPI. (C) Clusters of Orthologous Genes (COG) categories are indicated with their relative abundance for the same genes showing either decreased (orange) or increased (green) fitness at 24 h HPI (light shade), 48 h HPI (dark shade), and both (hatched).

Initial attempts to produce *Krmit* insertion tags directly from homogenized infected tissues were unsuccessful due to the inability to extract non-sheared GAS gDNA required for Tn-seq. Consequently, homogenized tissue lysates were grown in THY broth for 4 h at 37°C to allow for a limited expansion of the mutant libraries (*ca*. 5–6 generations). GAS cells were collected after outgrowth, and *Krmit* insertion tags produced to allow for deep sequencing (see Material and methods). The ratios of mutant abundance were calculated for each GAS 5448 gene (log_2_FC) comparing the output at 12, 24 and 48 HPI to the input (T_0_) mutant pools to identify potential subcutaneous fitness genes (*scf*). At 12 HPI, the *Krmit* library composition remained relatively unchanged compared to the initial input library (Fig 4A, S2 Table), with only 6 genes identified (3 for increased, 3 for decreased fitness). Increasingly more substantial changes were observed at 24 then 48 HPI (Fig 4B and 4C, S2 Table). We found 75 (24 h) and 106 (48 h) genes associated with decreased *in vivo* fitness, with 34 common to both time points (Fig 5A). We also identified 29 (24 h) and 107 (48 h) genes linked to increased *in vivo* fitness, with 10 of those common to both time points (Fig 5B).

COG enrichment analyses of the *in vivo* Tn-seq dataset revealed that the most represented category for genes linked to increased (55 of 147, 37%) or decreased (53 of 126, 42%) fitness at all time points was that of "unknown function" (Fig 5C). Of the remaining genes found at 24 and/or 48 HPI associated with decreased *in vivo* fitness, transport and metabolism of amino acids (~10%) and inorganic ions (~8%) were highly represented (Fig 5C). For genes linked to increased *in vivo* fitness, transport and metabolism of carbohydrates (~10%) and DNA replication (~8%) appeared to be limiting fitness in this environment (Fig 5C). When the Tn-seq dataset obtained for M1T1 GAS 5448 *in vitro* fitness in THY was compared to the *in vivo* results, 41 genes were found in both screens (S3 Table). This included 18 (decreased) and 9 (increased) genes with correlating phenotypes in both screens, while 14 genes presenting opposite phenotypes *in vitro* and *in vivo*.

### Validation of the *in vivo* Tn-seq screen

Insertional inactivation mutants were generated in 6 genes associated with decreased fitness *in vivo* (RS02090/*lytR*, RS02780 /*yvqE*, RS04065/*dltA*, RS06590/*adcA*), RS06895 and RS08410/*mga*) and tested individually against WT 5448 using an *in vivo* competitive infection assay in the murine skin and soft tissue model (see Material and methods). CD1 mice were subcutaneously infected with *ca*. 1:1 ratios of WT and each mutant, and population composition monitored after plating excised lesion lysates for CFU counts after 48 HPI.

**Fig 6:**
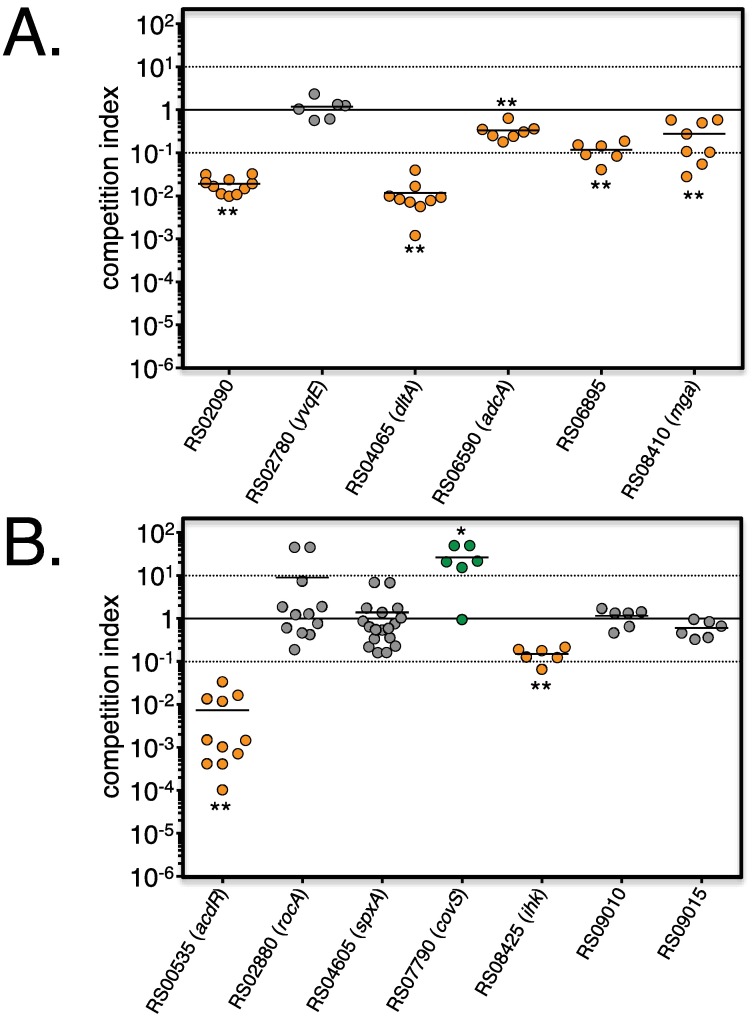
Validation of the *in vivo* Tn-seq screen. Insertional mutants in selected genes identified by Tn-seq as associated with decreased (A) or increased (B) fitness *in vivo* in lesions were mixed with GAS 5448 (*ca*. 1:1 ratio) and monitored during competitive infection during soft tissue infection of CD1 outbred mice. Total CFU were determined from homogenized lesions harvested at 48 HPI and ratios expressed as competitive indexes (mut/wt). Results correlating with the *in vitro* Tn-seq screen for decreased (orange circles) and increased (green circles) fitness are shown. Results not validating Tn-seq data are displayed as grey circles. Unpaired student’s *t*-test was used to evaluate the significance of differences between groups; a *p* value of <0.05 (*) or <0.01 (**) was considered statistically significant.

Of the 6 tested mutants in genes linked to decreased fitness, 5 (*lytR*, *dltA*, *adcA*, RS06895 and *mga*) were outcompeted by 5448 in the lesions with a competitive index less than 1 (Fig 6A), strongly supporting the *in vivo* Tn-seq data. Only *yvqE* did not show any competitive defect in the assay, in contrast to the *in vivo* Tn-seq screen (Fig 6A). However, as mutants in *yvqE* showed an *in vitro* fitness defect (Fig 2A, S1 and S3 Tables), this likely represents a false positive in the *in vivo* Tn-seq screen due to the 4 h *in vitro* library expansion. In contrast, *mga* showed opposite phenotypes *in vitro* (increased fitness) and *in vivo* (decreased fitness) (S3 Table). Competitive assays confirmed that *mga* was critical during subcutaneous infection as seen in the *in vivo* Tn-seq (Fig 6A), but this is likely an underestimation given its potential recovery during *in vitro* expansion (Fig 2A).

Selected mutants in genes associated with increased fitness *in vivo* (RS00535/*adcR*, RS02880/*rocA*, RS04605/*spxA1*, RS07790/*covS*, RS08425/*ihk*, RS09010 and RS09015) were also assayed by *in vivo* competition against WT 5448 (Fig 6B). Only 1 out of the 7 mutants tested (*covS*) recapitulated the *in vivo* Tn-seq findings, although *rocA*, which is associated with the *covS* genetic switch, trended upwards. Three of the tested genes (*ihk*, RS09010 and RS09015) were chosen as they were also found in the *in vitro* Tn-seq screen (S1 and S3 Tables) with 2 (RS09010 and RS09015) that were experimentally validated (Fig 2B); strongly suggesting that RS09010 and RS09015 correspond to false positive in the *in vivo* Tn-seq screen. Despite our pilot experiment finding pre-existing *Krmit* insertions in *covS* linked to mucoid colonies (S3 Fig), we cannot rule out that spontaneous mutations in *covS* and possibly *rocA* are occurring *in vivo* in mutants containing unlinked *Krmit* insertions. This suggests that mutations conferring a selective advantage for GAS *in vivo* or during the *in vitro* outgrowth step can bias the Tn-seq findings and emphasizes the importance of independent validation.

### *scfAB* encode proteins of unknown function critical for M1T1 GAS 5448 pathophysiology

**Fig 7:**
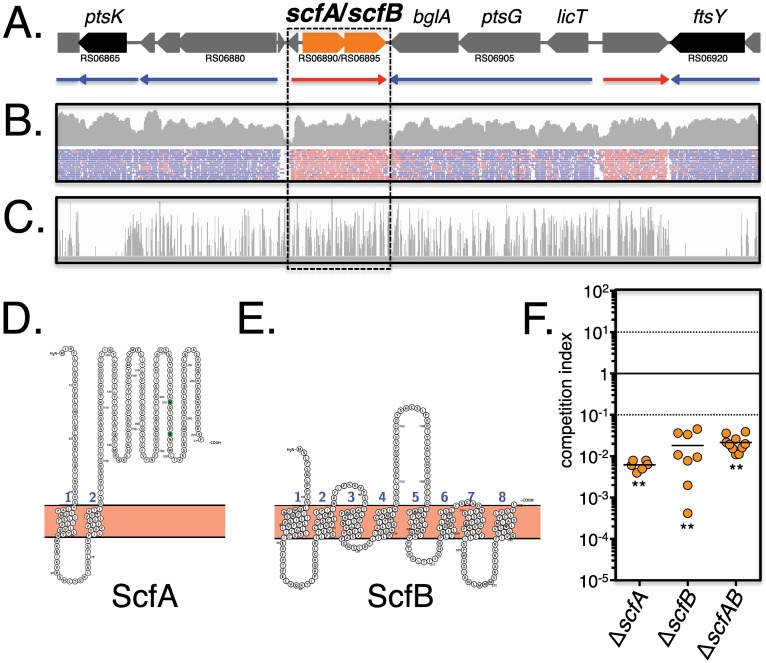
The *scfAB* operon is dispensable *in vitro*, but is required for *in vivo* fitness. (A) The M1T1 GAS 5448 genomic organization of *scfAB* (orange) with surrounding non-essential (grey) and essential (black) genes are shown with predicted transcriptional units from RNA-seq from (B) below with red (+ strand) and blue (- strand) arrows. (B) RNA-seq for the M1T1 GAS 5448 genomic region surrounding *scfAB* at late log phase in THY as displayed in IGV. RPKM (upper panel) and reads (lower panel) are shown with direction indicated as in (A). (C) Tn-seq analyses of the same genomic region after growth in THY and displayed in IGV. Height of the bars indicates the number of *Krmit* insertions mapping to that location. (D and E) Protein topology models of ScfA and ScfB (Protter), respectively, indicating membrane localization with 8 (ScfA) and 2 (ScfB) predicted transmembrane domains. (F) Non-polar mutants in the *scfAB* locus (Δ*scfA*, Δ*scfB*; and Δ*scfAB*) were mixed with parental GAS 5448 (*ca*. 1:1 ratio) and monitored during competitive soft tissue infection of CD1 outbred mice. Total CFU were determined from homogenized lesions harvested at 48 HPI and ratios expressed as competitive indexes (mut/wt). Unpaired student’s *t*-test was used to evaluate the significance of differences between groups; a *p* value of <0.05 (*) or <0.01 (**) was considered statistically significant.

The *in vivo* Tn-seq screen identified RS06895 as critical during GAS 5448 fitness during subcutaneous infection at 24 h (S2 Table); and *in vivo* competitive infection assays conducted using a RS06895 *Krmit* mutant validated this finding (Fig 6A). Interestingly, the adjacent RS06890 gene was also shown to be necessary for *in vivo* fitness at 48 HPI (S2 Table). RNA-seq analyses performed on GAS 5448 grown in THY to exponential phase indicate that RS06890 and RS06895 likely form a bicistronic operon (Fig 7A and 7B). Importantly, both genes were dispensable during *in vitro* growth (Fig 7C, S1 Table), thus targets for knockout mutagenesis. RS06890 and RS06895 were renamed *scfA* and *scfB*, respectively, and subjected to further analysis.

The *scfA* and *scfB* genes are found within the GAS core genome [43] and both are annotated to encode for hypothetical membrane spanning proteins of unknown function. Using the topology prediction tool Protter [50] to analyze ScfA (300 AA; pI 8.20; Mw: ~33 kDa) identified eight putative transmembrane domains (Fig 7D, 1 to 8) and seven exposed domains (Fig 7D, a to g). Analyses of ScfB (271 AA; pI 7.77; Mw: ~31 kDa) indicated two possible transmembrane segments (Fig 7E, 1 and 2) and three projecting domains (Fig 7E, a to c). Homologs of GAS *scfAB* were found as linked genes of unknown function in the genomes of 21 *Streptococcus*, *Enterococcus* and *Bacillus* species, with the surrounding genetic context varying extensively (S4 Fig). In *S*. *mutans*, a transposon screen found that the homologs SMU.746 (*scfA*) and SMU.747 (*scfB*) were important for the acid stress response and biofilm formation [51]. Although the authors hypothesized that the genes encoded for an amino acid permease, that was not demonstrated [51]. In *B*. *subtilis*, expression of the uncharacterized *ycgR* (*scfA*) and *ycgQ* (*scfB*) is controlled by ECF σ factors involved in environmental stress response and cell envelope homeostasis [52]. In GAS, *scfA* was previously identified as required for M1T1 GAS 5448 survival in human blood in our TraSH screen [39], suggesting a role in disseminated GAS infection.

**Fig 8:**
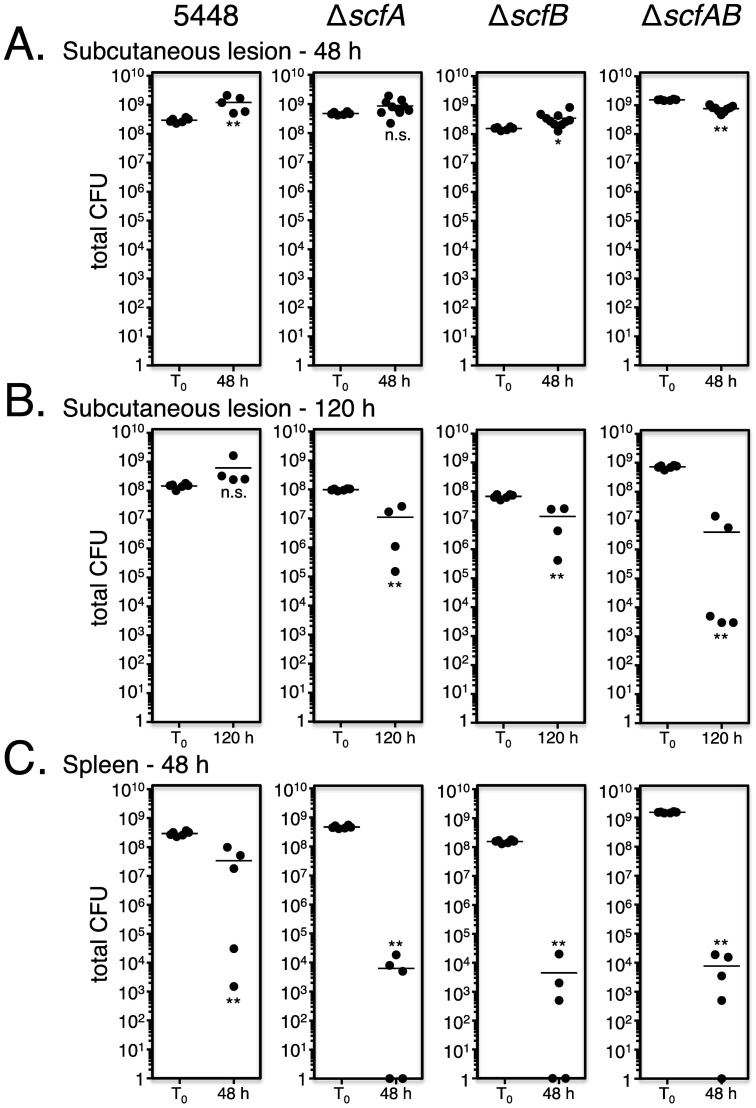
*scfAB* is required for soft tissue infection and dissemination. Pure cultures of GAS 5448 and isogenic Δ*scfA*, Δ*scfB* and Δ*scfAB* mutants were individually inoculated (T_0_) subcutaneously into CD1 mice and total CFU counts were determined from lesions excised at 48 (A) and 120 (B) HPI to monitor infection, as well as from spleens harvested at 48 HPI (C) to assess dissemination compared to wild type GAS 5448. Unpaired student’s *t*-test was used to evaluate the significance of differences between groups; a *p* value of <0.05 (*) or <0.01 (**) was considered statistically significant.

Non-polar mutations of *scfA*, *scfB*, and both *scfAB* in M1T1 GAS 5448 were constructed by allelic exchange and verified by qRT-PCR (S5 Fig). The resulting mutants (Δ*scfA*, Δ*scfB* and Δ*scfAB*) were tested against WT 5448 in *in vivo* competitive assays in the CD1 mouse subcutaneous infection model (see Material and methods). All three mutants were strongly outcompeted by WT 5448 *in vivo* after 48 HPI (Fig 7F). To assess whether the individual *scfAB* mutants were attenuated *in vivo*, each was used as pure culture to infect CD1 mice through the subcutaneous route and total bacterial burden (CFU) in lesions was compared to WT 5448 infection at 48 and 120 HPI. CFUs were similar to the infecting dose (10^8^−10^9^) after 48 HPI for all the tested GAS strains (Fig 8A). Although the cell counts at 120 HPI increased in the lesion infected with WT 5448, they decreased significantly for Δ*scfA* (1-log), Δ*scfB* (1-log), and Δ*scfAB* (2-log) (Fig 8B). Dissemination of GAS was also assessed by determining bacterial burden in the spleen at 48 HPI, and an approximate 5-log reduction in CFU was observed for Δ*scfA*, Δ*scfB* and Δ*scfAB* compared to WT 5448 (Fig 8C). Altogether, our results revealed that the conserved *scfAB* operon encodes putative membrane proteins that are critical for GAS 5448 during the initial subcutaneous infection as well as for dissemination into the bloodstream during invasive infection. Furthermore, our *in vivo* Tn-seq dataset allows us to assign functional information for M1T1 GAS genes previously annotated as "unknown function".

## Discussion

The Group A Streptococcus (GAS) remains a highly prevalent human pathogen capable of infecting multiple niches within its human host. Common to all GAS infections are the steps of initial colonization of an epithelium (throat or skin) and subsequent propagation to the subepithelial tissues [8]. In this report, we used the clinically relevant M1T1 GAS 5448 to perform the first *in vivo* Tn-seq screen to characterize, on a whole-genome level, those GAS genetic determinants (subcutaneous fitness genes, *scf*) functionally required within the subepithelial niche, as well as mutations that could potentially confer a selective advantage during the infection process. Although many were annotated with specific roles, we found that a substantial portion of the identified *scf* genes were of unknown function. Of these, we selected two undetermined genes, *scfA* and *scfB*, present in the GAS core genome and confirmed their role *in vivo* during soft tissue infection and GAS dissemination into the bloodstream.

### Tn-seq to interrogate GAS pathophysiology *en masse*

High throughput Tn-seq screens (also TraDIS, HITS or IN-seq) [42, 53–55] take advantage of massive parallel DNA sequencing to monitor in a qualitative (nucleotide-level insertion location) and quantitative (mutant prevalence and gene fitness index) manner the composition of complex mutant pools [56–58]. These approaches permit biosystems-level analyses of bacterial genomes to accelerate functional genomics [20]. Depending on the experimental settings used (*in vitro*, *ex vivo* or *in vivo*), Tn-seq has helped define bacterial minimal genomes (*i*.*e*., essential genes) [42, 53–55], cell responses to environmental stresses [59, 60] or to antibiotics [61] and bacterial pathogenesis [62–65].

Tn-seq and other competition-based genetic screens (TraSH, STM) using complex mutant libraries do have certain limitations that prevent certain classes of genes from being identified. Classically, secreted and surface-associated molecules can be provided through *trans*-complementation by others in the library and mask the phenotype of that genes mutant in the library. For GAS virulence in soft tissue investigated here, important virulence factors such as toxins, superantigens, proteases, DNases, and capsule were not be expected in our screen likely due to this issue. In contrast, this approach may favor the discovery of important metabolic and regulatory pathways that are confined to the cytoplasm of GAS.

Our group has developed *mariner*-based genetic tools [38] to accelerate GAS functional genomic analyses through *en masse* TraSH [39] and Tn-seq [43] screens. Using two divergent GAS strains (M1T1 5448 and M49 NZ131), we recently conducted *in vitro* Tn-seq screens to characterize the GAS minimal core genome [43], defining potential targets for new therapeutics. GAS 5448 was isolated from a patient with necrotizing fasciitis (NF) and toxic shock syndrome [66] and is a representative of the globally disseminated M1T1 serotype that has been extensively used to study GAS pathogenesis in mice [39, 47, 67, 68]. Moreover, its genome sequence was recently completed [37]. For our essentiality studies, we generated a highly complex transposon mutant library in GAS 5448 containing over 85,000 unique *Krmit* insertions [43] that represented the baseline mutant pool used in this work. In our initial qualitative analyses of this 5448 *Krmit* library using a Bayesian statistical model [43], we predicted that 227 genes were essential (non mutable) and therefore indispensable *in vitro*. Such genes containing very few or no insertions are, by their nature, much more difficult to track during quantitative (*i*.*e*., fitness) Tn-seq analyses, and were not considered in our current study.

Here, the remaining dispensable genes were initially interrogated by Tn-seq for more subtle phenotypes conferring decreased or increased fitness *in vitro*. We monitored the composition of the GAS 5448 *Krmit* pool *en masse* to determine differential mutant abundance using DEseq2 [69] and EdgeR [70], two bioinformatics pipelines originally designed for RNAseq analyses. Other Tn-seq analysis tools are now available, such as MAGenTA [71, 72], which can track all individual insertions and generate fitness values for each insertion (nucleotide resolution). However, DEseq2 and EdgeR have recently gained acceptance for Tn-seq gene fitness analyses based on their availability and ease-of-use [58, 60, 73–75]. We compared the two pipelines by re-analyzing the datasets obtained for the GAS 5448 *Krmit* mutant libraries after two 24 h passages in THY rich medium [43] to compare mutant pool composition over time and selected DEseq2 for our analyses as it produced more conservative datasets.

A total of 139 genes associated with decreased GAS 5448 fitness *in vitro* were identified at the 24 h or 48 h time points using DEseq2, with 38 genes common to both (S1 Fig, S1 Table). Using the Bayesian statistical analysis, we had categorized genes as “critical” (n = 71) that were found essential only after 2 *in vitro* passages in THY and we proposed they might represent genes important for *in vitro* fitness. However, out of the 139 genes found linked to decreased fitness *in vitro* by DEseq2 (S1 Fig, S1 Table), only 22 were also found critical by Bayesian analysis [43] (Fig 1B and 1C, orange diamonds). This discrepancy is not surprising as the mode of data analyses between these two tools is quite different. Bayesian analysis takes into account the library coverage (*Krmit* positions) regardless of read depth (counts) and identifies significant gaps in the genome with no transposon insertions; therefore, a gene is essential if a portion of the gene (*e*.*g*., essential domain) lacks insertions. DEseq2 will miss this gene as it monitors the read counts for the entire gene length, and data integration would likely mask the results for the essential domain.

Data validation by *in vitro* competition growth assays confirmed 4 out of the 5 genes (80%) associated with decreased fitness that we tested (Fig 2A). Of the 98 mutations conferring increased *in vitro* fitness by DEseq2, validation experiments confirmed only 3 out of the 5 tested mutations (60%) (Fig 2B), revealing a slightly higher proportion of false-positive results with genes in this category. In general, false-positive results are not uncommon with Tn-seq screens [58, 64, 76] and may reflect a variety of reasons. The initial Tn-seq screen is a complex competitive environment involving thousands of mutants whereas validation studies tend to compare only a single mutant to the WT pathogen. Also, the defined mutant generated for validation are likely to differ genetically from the original transposon mutations found in the complex library. Genetic manipulation of GAS 5448 can be difficult and we used mutants produced by stable plasmid insertional inactivation instead of allelic exchange for our validation tests; a strategy that could create (i) a polar effect on adjacent genes when in operon or (ii) a truncated allele of the target gene that could still be functional. Overall, we were still able to validate 70% of the genes tested (Fig 2).

### M1T1 GAS 5448 genetic determinants impacting fitness during subcutaneous infection

For our *in vivo* Tn-seq screen of GAS pathophysiology, we selected a subcutaneous infection model in immunocompetent Crl:SKH1-hrBR mice that displayed symptoms with the *Krmit* library similar to those observed with wild type GAS 5448 (S2 Fig) and supported high *Krmit* mutant counts (> 10^8^ CFU) (Fig 3) allowing for pool complexity during infection. Our *in vivo* screen identified 147 genes (with 75 and 106 genes at 24 and 48 HPI, respectively) as potentially critical for the M1T1 GAS 5448 fitness during subepithelial infection. As observed for our *in vitro* fitness analysis, the most prevalent COG category (55 genes, 37%) was annotated as encoding proteins of unknown function.

Although we could validate 5 out the 6 genes (80%) identified as linked to decreased *in vivo* fitness, a defined mutant in RS02780/*yvqE* exhibited no defect when competed directly with wild type GAS 5448 in mice (Fig 6A). Since a *yvqE* mutant also displayed decreased competitive fitness *in vitro* (Fig 2A), it likely leads to a false positive finding *in vivo*. This result highlights that the 4 h *in vitro* library expansion step in our *in vivo* Tn-seq screen setting likely introduces some bias into our *in vivo* fitness datasets. Mutant pool expansion following *in vivo* Tn-seq screens, by mutant pool plating on agar [65, 76, 77] or culture in broth [64, 78], is often required when library DNA extraction directly from tissue represents a limiting step to the Tn-seq procedure. This extra step is particularly important when using the *Mme*I-based Tn-seq approach [72], as it requires high quality DNA (no shearing). In this case, our data revealed that it is important to take into account the competition phenotypes from both *in vivo* and *in vitro* validation experiments to distinguish phenotypes truly displayed *in vivo*.

Several established genes were found to be critical in the subepithelial environment, including a subunit of the D-alanine-activating enzyme DltA (RS04065/*dltA*) involved in the biosynthesis of D-alanylated lipoteichoic acid in GAS. A GAS 5448 *dltA* mutant was previously found to have diminished ability to adhere and invade human pharyngeal epithelial cells [79] and was attenuated for survival in whole human blood and neutrophils [80]. RS02090/*cpsA*, encoding a LytR-like transcriptional regulator known to regulate capsule expression, cell wall homeostasis and virulence in *Streptococcus agalactiae* (Group B Streptococcus, GBS) [81, 82], was also identified as required for GAS fitness *in vivo*. Our findings differ from published reports showing that a *cpsA* (*lytR*) mutant was more virulent than WT in subcutaneous infection [83]. However, these studies used an invasive M1 *covS* strain GAS 1529, suggesting that loss of LytR (CpsA) could increase GAS fitness in a *covS*^-^ background.

RS06590/*adcA*, encoding a Zn-specific transporter [84], was also linked to *in vivo* fitness. Homologs of *adcA* have been found in other pathogenic streptococci, where it is typically found associated with an operon *adcCBA*. In GBS, AdcA was found to be required for Zn acquisition, proper cell morphology, and fitness in human amniotic fluid and cerebrospinal fluid [85]. In *S*. *gordonii*, *adcA* is important for manganese homeostasis, biofilm formation and natural competence [86, 87], while in *S*. *pneumoniae adcA* is critical for natural competence [88], fitness in human serum [89]; and virulence [89, 90]. Our study and others finding *adcA* important *in vivo* supports the hypothesis that zinc homeostasis is critical during multiple steps of GAS infection [39, 91].

The *in vivo* Tn-seq screen also identified *mga* as critical during GAS 5448 subepithelial infection, which is consistent with previous observations showing that a *mga* mutation led to virulence attenuation in different *in vivo* and *ex vivo* GAS infection models [11, 39, 92–95]. Interestingly, *in vitro* Tn-seq revealed two opposite phenotypes whereby inactivation of *mga* resulted in increased GAS 5448 fitness in THY (Fig 2B). This suggests that the *mga*-associated *in vivo* phenotype would be even stronger, except that the 4 h library expansion restored some of the *in vivo* reduction. Mga is an important PTS-regulatory domain (PRD)-containing virulence regulator (PCVR) in GAS that controls the expression of the virulence factor M protein [11]. However, the *emm* gene was not identified in the *in vivo* Tn-seq screen, suggesting that the *mga* phenotype observed in the subepithelial environment might not be related to *emm* expression. We are currently pursuing the exact role that Mga plays in M1T1 GAS 5448 fitness in this *in vivo* niche.

Our Tn-seq screen also identified 126 genes linked to increased *in vivo* fitness at 24 or 48 HPI, with 10 genes common to both. Interestingly, validation by *in vivo* competitive infection found that only 1 out of the 7 defined mutants tested (*covS*) recapitulated the *in vivo* Tn-seq findings (Fig 6B). Thus, *in vivo* Tn-seq demonstrated that *covS* mutants, and possibly *rocA* mutants through activation of *covS*, confer a fitness advantage in M1T1 GAS leading to selection in the subepithelial tissues. This reveals another potential technical bias that might be occurring during the *in vivo* Tn-seq whereby a *covS* mutation occurs spontaneously in the *Krmit* mutant pool independent from pre-existing *covS Krmit* insertions. This would result in genes found by Tn-seq associated with increased *in vivo* fitness that fail to validate. Moving forward, we will modify our *in vivo* Tn-seq screen and data analyses to take into account spontaneous *covS* mutations on M1T1 GAS 5448 fitness based on Tn-seq from near saturation *Krmit* libraries in the isogenic 5448AP (*covS*^-^) strain.

Of course, lack of validation may simply reflect differences between the defined and library mutants as for the *in vitro* validation or represent false positives (*yvqE*, RS09010, RS09015, and RS08425/*ihk)* based on the *in vitro* Tn-seq datasets. Although RS00535/*adcR* was initially identified as linked to increased *in vivo* fitness, our validation revealed that an *adcR* mutant was attenuated *in vivo*, which is consistent with observations by Sanson *et al*. [96]. Interestingly, AdcR can activate *hasABC* [96], the same capsule operon that is derepressed during the *covS* switch.

### Role of ScfAB in subepithelial invasion and dissemination

Tn-seq screens represent a powerful approach to accelerate bacterial genomics, particularly by providing functional annotations for genes encoding proteins that are either poorly characterized or of unknown function [20, 58]. The most prevalent COG category of subcutaneous fitness (*scf*) genes found in our *in vivo* Tn-seq screen (108 genes), both for increased and decreased fitness, were those of "unknown function". We decided to focus on two adjacent genes, RS06890 (*scfA)* and RS06895 (*scfB*) that were critical for *in vivo* fitness as representative of this group. Analyses of defined mutants in this locus revealed that *scfAB* were in an operon that was important at the subepithelial site of infection as well as for GAS 5448 dissemination into the bloodstream (Figs 6–8). Both genes encode for putative transmembrane proteins and BLAST analyses did not reveal any homologs where a function was available. The *scfAB* operon is part of the GAS core genome and both genes appear conserved in GAS. Studies are underway to investigate the role of the *scfAB* operon during infection at different infection sites and in different GAS strains to see if their function is ubiquitous or strain-specific. Homologs of *scfAB* are found in other pathogenic streptococci and closely-related Gram-positive pathogens (S4 Fig); however, the locus has only been studied in one report. In *S*. *mutans*, *scfA* and *scfB* homologs were found in a transposon mutagenesis screen to impact acid stress response and biofilm formation [51, 52]. Although the authors proposed that they encoded amino-acid transporters, no further analysis was performed. Thus, *scfA* and *scfB* appear to be ubiquitous in other pathogenic streptococci and Gram-positive pathogens and further analyses are warranted to better understand their cellular function. Importantly, our *in vivo* Tn-seq was key in providing an *in vivo* phenotype for genes encoding "unknown function" proteins and has the potential to enhance our annotation of the GAS genome and the identification of novel therapeutic targets.

### Concluding remarks

This work represents the first *in vivo* Tn-seq screen to functionally interrogate the GAS genome in the context of a disease-relevant environment. The identification of 147 genes potentially critical *in vivo* reveals that adaptation to *in vivo* niches is a complex process involving multiple aspects of the GAS cell physiology (metabolism, gene regulation, cell envelop). We are now investigating the genetic requirements of the M1T1 GAS 5448, and its counterpart 5448AP, using multiple infection models *in vivo*, *ex vivo* and *in vitro*. In addition, we have a rich dataset of genes with predicted function as well as those previously annotated as "of unknown function" that can be further mined to discover new aspects of GAS pathophysiology. We have also established complex *Krmit* libraries in additional GAS strains to help study the effect of genome variation on GAS pathogenesis.

## Materials and methods

### Bacterial strains and media

Bacterial strains used in this study are listed in S4 Table. GAS 5448 is a representative of the globally disseminated invasive serotype M1T1 clone isolated from a patient with necrotizing fasciitis and toxic shock [66]. The *Krmit* mutant library in GAS 5448 was produced by *in vivo mariner* mutagenesis [43] and contains over 85,000 independent *mariner* transposants (*i*.*e*., one *Krmit* TIS for every 22 nucleotides). GAS strains were routinely cultured in Todd-Hewitt medium (Alpha Biosciences) supplemented with 0.2% yeast extract (THY) as described [97]. *Escherichia coli* strain DH5α was routinely used as host for plasmid construction and preparation and cultured in Luria-Bertani (LB) medium (EMD Chemicals). Antibiotics (Gold Biotechnology) were used at the following concentrations: Spectinomycin (Sp) at 100 μg/ml for both *E*. *coli* and GAS, and Kanamycin (Km) at 50 μg/ml for *E*. *coli* and 300 μg/ml for GAS.

### Molecular genetics

Oligonucleotides used in this study were synthesized by Integrated DNA Technologies, Inc. and are listed in S5 Table. Plasmids used in this study are presented in S4 Table. Plasmids were isolated using the Wizard *Plus* SV Minipreps kit (Promega) or the QIAGEN Plasmid Purification Midi Kit (QIAGEN). Restriction enzymes, Antarctic Phosphatase and T4 DNA ligase (New England Biolabs) were used according to the manufacturer’s instructions. PCR was performed using either *Taq* DNA polymerase (New England Biolabs) or High-Fidelity AccuPrime *Pfx* DNA polymerase (Life Technologies) with 1 μg of DNA template and 10 pmol of the appropriate primers (S5 Table). Arbitrary-primed-PCR (AP-PCR) experiments were conducted as previously described [38]. When necessary, PCR products were purified using Wizard SV Gel and PCR Clean-Up System kit (Promega). Transformations were performed with the Gene Pulser Xcell System apparatus (Bio-Rad) as recommended by the manufacturer, using electrocompetent cells of *E*. *coli* or GAS prepared as previously described [38, 98]. Genomic DNA (gDNA) from GAS was purified using the MasterPure Complete DNA Purification kit (Epicentre Biotechnologies). Sanger DNA sequencing was performed by Genewiz, Inc.

### Gene insertional inactivation

When available, *Krmit* transposon mutants in GAS 5448 were selected (S4 Table). Mutants 5448KM07790, 5448KM08410, 5448KM09015 and 5448KM09010 present a *Krmit* integrated in the gene RS07790/*covS* [TA at position 211 within the ORF (TA_211_)], RS08410/*mga* (TA_4_), RS09015 (TA_412_) and RS09010 (TA_471_), respectively. Additional mutant strains in GAS 5448 (S4 Table) were produced using the pSinS/pHlpK system [43]: succinctly, an internal fragment of the gene of interest was amplified by PCR using the appropriate primers (S5 Table) and the resulting DNA fragment cloned into the *BamHI* site of the pSinS suicide vector. The resulting recombinant plasmid (S4 Table) was then transformed into GAS 5448 cells containing the pHlpK helper plasmid and mutagenesis carried out as previously described [43]. Plasmid integration within the targeted gene was verified by PCR using the appropriate primer(s) (S5 Table). For simplification in the Result section, insertional mutant strains were referred to by the name of the mutated gene (*e*.*g*., mutants 5448KM07790 and 5448ii01780 were designated RS07790 and RS01780, respectively).

### *In vitro* competition growth assay

Competition growth assays in THY were carried after inoculation of 10 ml of broth with comparable amounts of exponentially-grown cells of GAS 5448 and the tested mutant strain (close to a 1:1 ratio). At different time intervals (24, 48, 72h), samples were collected, serial diluted (10-fold increments), cell suspension plated on either THY (whole population) or THY containing the appropriate antibiotic (mutant population) and cell counts determined. Competition index (CI) was calculated using the following formula: CI = (R_M_/R_W_)/(R_M0_/R_W0_), with R_M0_ and R_W0_ correspond to the ratio of the mutant and the ratio of the wild-type, respectively, in the initial inoculum (T_0_); and R_M_ and R_W_ correspond to the ratio of the mutant and the ratio of the wild-type, respectively, at the end of the competition growth assay. Unpaired student’s *t*-test was used to evaluate the significance of differences between groups; a *p* value of <0.05 was considered statistically significant.

### Ethics Statement

Crl:SKH1-*hr*BR mice (Charles River Laboratories) were infected with GAS in AAALAC-accredited ABSL-2 facilities and following protocols approved by the University of Maryland IACUC (R-16-05) for humane treatment of animal subjects in accordance with guidelines set up by the Office of Laboratory Animal Welfare at NIH, Public Health Service, and the Guide for the Care and Use of Laboratory Animals; with every effort to limit distress and pain to animals taken.

### Model of GAS 5448 subcutaneous infection suitable for Tn-seq

Overnight cultures of GAS 5448 or the GAS 5448 *Krmit* mutant library were diluted 1:20 to a final volume of 80 ml of THY broth and grown to late-logarithmic phase, GAS cell chains were disrupted by vortexing the culture for 10 min, and cells centrifuged at 6000 × *g* for 10 min and resuspended in saline to produce the infection inoculum (*ca*. 10^9^ CFU/ml). Initial CFU counts of the infectious dose were confirmed by serial dilutions plated onto THY agar (THYA) plates. Five-week-old, outbred, immunocompetent, hairless female Crl:SKH1-*hr*BR mice (Charles River Laboratories) received subcutaneous injections of *ca*. 10^8^ CFU (0.1 ml) of 5448 (or 5448 *Krmit* mutant library) at the base of the neck. At different times (t = 12, 24, 48 hours post infection), mice were euthanized by CO_2_ asphyxiation and skin lesions were excised.

For microscopy analyses, lesions were fixed with fresh 4% paraformaldehyde for 24 hours at 4°C, stored in 70% ethanol at room temperature, then paraffin embedded for sectioning and stained using a hematoxylin and eosin stain (H&E stain) for histology observations.

To quantify bacterial load, the lesion was placed into a sterile 2-ml screw-cap microtube containing 1.4 mm ceramic spheres (Lysing Matrix D, MP Biomedicals) and 1 ml of sterile saline; and skin tissues were homogenized using three successive 45-second bursts with a FastPrep FP120 BeadBeater (BioSpec Products) as recommended by the manufacturer. Tissue homogenates (3 ml final) were serially diluted (10-fold increments) in saline, plated onto THY agar, and CFU counts determined after overnight incubation at 37°C.

### *In vivo* Tn-seq analyses

Tissue homogenates (3 ml final) were produced as described above using the skin lesions from mice infected with the GAS 5448*Krmit* mutant library and were transferred into 150 ml THY+Km and cultured at 37°C for 4 h. GAS cells were collected by centrifugation at 6000 × *g* for 10 min and GAS gDNA extracted as above. Tn-seq was carried using the *Mme*I protocol [42] with modifications described by Le Breton *et al*. [43]. In this study, twelve different *Mme*I adapters (S5 Table) were used to allow sample multiplexing on Illumina lanes. Libraries of *Krmit* insertion tags were sequenced (50-nt single end reads) on an Illumina HiSeq 1500 platform in the Institute for Bioscience and Biotechnology Research (IBBR) Sequencing Facility located at the University of Maryland, College Park. Tn-seq read datasets were analyzed (quality, filtering, trimming, alignment, visualization) as previously described [43] using the GAS 5448 genome [37] for read alignments. A more detailed description of the bioinformatics analyses is provided in S1 Text. The ratios of mutant abundance comparing the output to input mutant pools were calculated as a fold change (FC) for each GAS gene using the DEseq2 and EdgeR pipelines [69, 70].

### Inactivation of *scfA*, *scfB*, and *scfAB* in GAS 5448

Nonpolar mutations in the *scfAB* locus were obtained by replacing the corresponding open reading frames (ORF) with the promoterless *aphA3* gene using allelic exchange as previously described [38]. Primers, plasmid constructs and GAS strains are listed in S4 and S5 Tables. For the *scfA* mutation, DNA fragments flanking the *scfA* gene were amplified using the primer pairs oAX0478.1 and oAX0478.2 (before the 5′ end of *scfA*) (PCR 5’*scfA*) and oAX0478.3 and oAX0478.4 (within the 3′ end of *scfA*) (PCR 3’*scfA*) and subsequently ligated by PCR-splicing by overlap extension (SOE) to the *aphA3* cassette. The resulting PCR product then was digested by *BamHI* and cloned into the *BamHI*-digested pCRS [38], creating the plasmid pAX0478K (S4 Table). Allelic replacement of the *scfA* by the *aphA3* cassette was conducted as previously described [38], creating the GAS strain 5448Δ*scfA*. The same approach was carried out to mutate *scfB* using *scfB* flanking DNA fragments amplified with primers oAX0477.1 and oAX0477.2 (PCR 5’*scfB*) and oAX0478.3 and oAX0478.4 (PCR 3’*scfB*) to produce the pAX0477K plasmid and generate GAS strain 5448Δ*scfB* (S4 and S5 Tables). Expression of *scfA* and *scfB* in strains 5448Δ*scfA* and 5448Δ*scfB* was verified by qRT-PCR as previously described [40] using the appropriate primers (S2 Table). To generate the GAS strain 5448Δ*scfAB*, the *aphA3* cassette was linked to PCR products PCR 5’*scfA* and PCR 3’*scfB* to create the plasmid pAX0478-77K (S4 and S5 Tables).

### *In vivo* competition growth assay

Subcutaneous infections were carried out using 5- to 6-week-old female CD-1 mice (Charles River Laboratories). Cell suspensions of exponentially growing GAS cells were obtained by mixing equal amounts of GAS 5448 and the tested mutant strain (close to a 1:1 ratio) in saline (*ca*. 10^9^ CFU/ml). Mice were anesthetized with ketamine, fur was removed from a ~3-cm^2^ area of the haunch with Nair (Carter Products), and 100 μl of a cell suspension in saline injected under the back skin. Mice were monitored twice daily for 2 days, euthanized by CO_2_ asphyxiation. Skin lesions lysates, CFU counts (in total population and mutant population) and competition indexes were obtained as described above. Unpaired student’s *t*-test was used to evaluate the significance of differences between groups; a *p* value of <0.05 was considered statistically significant.

### RNA-Seq and data analysis

GAS 5448 cells were grown to exponential phase in 20 ml of THY broth and treated with the RNAlater reagent (Qiagen). RNA extraction, RNA-seq library preparation and massive parallel DNA sequencing were carried out as previously described [40]. Read analyses were conducted as previously described [40] using the GAS 5448 genome for alignment [37]. Visualizations of the sequencing mapping were performed using the Integrative Genomics Viewer (IGV) [99].

### Infections with *scfA*, *scfB* and *scfAB* mutants

Cells suspensions in saline of pure cultures of GAS 5448, *scfA*, *scfB* and *scfAB* mutants were produced and injected subcutaneously in CD1 mice (10^8^−10^9^ CFUs) as described above. Mice were monitored twice daily for up to 5 days, euthanized by CO_2_ asphyxiation. Skin lesions and/or spleen were surgically harvested and cell lysates produced with the FastPrep FP120 BeadBeater and CFU counts determined as described above.

### Accession number for public deposition of RNA-seq and Tn-seq data

Illumina sequencing reads from the RNA-seq and Tn-seq analyses were deposited in the NCBI Sequence Read Archive (SRA) under the accession number (PRJNA391181).

## Supporting information

S1 Text Supplemental bioinformatics methods. (DOCX)

S1 Fig Comparison and classification of genes impacting M1T1 GAS M1T1 gene fitness during *in vitro* passage in THY. Venn diagrams comparing the number of genes showing either decreased (orange) or increased (green) fitness from the Tn-seq analyses of M1T1 GAS 5448 grown in THY for 24 h (A) and 48 h (B). (C) Clusters of Orthologous Genes (COG) categories are indicated with their relative abundance for the same genes showing either decreased (orange) or increased (green) fitness *in vitro* at 24 (light shade), 48 h (dark shade), and both (hatched).
(EPS)

S2 Fig Histopathology of M1T1 GAS 5448-infected lesions during murine skin and soft tissue infection. H&E stains of skin and associated soft tissues samples collected at 12, 24 and 48 HPI. Specific regions are indicated as follows: epidermis (EP), sebaceous gland (SG), hair shaft (HS), papillary dermis (PD), reticular dermis (RD), and panniculus carnosus muscle (PCM). (A) Non-infected tissues displaying normal epidermal thickness (*ca*. 12 μm) and features. (B) 12 HPI lesion with inflammatory infiltration (black arrows) in the dermis (both PD and RD) and PCM with early necrosis indicated. (C) Magnification of (B). (D) 24 HPI lesion with PCM inflammation and infiltration (black arrow); Hypo: hypodermis. (F) Magnification of (D) showing monocytes, macrophages, lymphocytes and neutrophils (blue, orange and green arrows, respectively) and visible GAS chains (black arrows). (E) 48 h HPI lesion showing extensive inflammation, tissue damage and abscess formation with pseudo-capsule (CAP) surrounding necrotic debris (Nec). (G) Magnification of (E) shows visible GAS chains (black arrows). Scale bars (in red) represent 100 μm on panels A, B, D and E; and 10 μm on panels C, F and G.
(EPS)

S3 Fig Genetic analyses of animal-passaged mucoid mutants. GAS 5448 *Krmit* libraries injected subcutaneously into immunocompetent Crl:SKH1-hrBR hairless mice were isolated from homogenized lesions and plated onto 5% TSA blood agar plates to look for mucoid (capsule-overproducing) colonies. (A) After 12 HPI, most colonies display a non-mucoid phenotype. (B) After 48 HPI, mucoid colonies (grey arrows) are distinguishable from the remaining colonies (white arrows). (C) AP-PCR analyses on gDNA isolated from selected mucoid mutants show *Krmit* insertions at different locations within the *covS* gene (green arrow heads) as indicated.
(EPS)

S4 Fig Comparative genome alignment for homologs of *scfAB* in Gram-positive bacteria. Genome alignment was generated using the Orthology Browser from the Microbes Online resource (www.microbesonline.org/) using the MGAS5005 genome as reference and the RS06890 homolog (M5005_Spy0477) as the query for the genome alignment. The graphic display presents genome alignments based on the 24 highest homologs to RS06890/M5005_Spy0477. Colored arrows depict homologous genes found within the display window of the different aligned genomes, while grey arrows represent genes that do not have a homolog in the displayed genome window.
(EPS)

S5 Fig The GAS Δ*scfA* and Δ*scfB* mutations are non polar. Real-time quantitative PCR (qRT-PCR) analyses of relative transcript levels for *scfA* and *scfB* genes in the Δ*scfA* and Δ*scfB* mutants compared to wild-type 5448 grown in THY to late log phase. Error bars represent the standard errors from at least three biological replicates. Dashed lines indicate 2-fold significance. Significance was determined using comparisons of transcript levels relative to those of the *gyrA* gene.
(EPS)

S1 Table Tn-seq analysis of the GAS 5448 genetic requirements for *in vitro* growth after 24-h and 48-h passages in THY broth medium. (XLSX)

S2 Table Tn-seq analysis of the GAS 5448 genetic requirements for *in vivo* growth during subcutaneous infections after 12, 24 and 48 HPI. (XLSX)

S3 Table Genes found during the *in vitro* (THY broth) and the *in vivo* (subcutaneous lesions) Tn-seq screens. (XLSX)

S4 Table Bacterial strains and plasmids. (PDF)

S5 Table Primers used in this study. (PDF)
