# Supplementary material for: Genome-wide discovery of novel M1T1 group A streptococcal determinants important for fitness and virulence during soft-tissue infection
Source: PLoS Pathog. 2017 Aug 23;13(8):e1006584. doi: 10.1371/journal.ppat.1006584 (PMC5584981; doi:10.1371/journal.ppat.1006584)
Supplement: S1 Text — (DOCX) [file ppat.1006584.s001.docx]

Supplemental Methods For:

**Genome-wide Discovery of Novel M1T1 Group A Streptococcal Determinants Important for Fitness and Virulence During Soft-Tissue Infection**

Yoann Le Breton^1^, Ashton T. Belew^1,2^, Jeffrey A. Freiberg^3^, Ganesh S. Sundar^1^, Emrul Islam^1^, Joshua Lieberman^4,*^, Mark E. Shirtliff^5,7^, Hervé Tettelin^5,6^, Najib M. El-Sayed^1,2^, and Kevin S. McIver^1^

^1^Department of Cell Biology & Molecular Genetics and Maryland Pathogen Research Institute, ^2^Center for Bioinformatics and Computational Biology, University of Maryland, College Park, College Park, Maryland, USA; ^3^Graduate Program in Life Sciences, ^4^Division of Infectious Diseases, ^5^Department of Microbiology and Immunology, ^6^Institute for Genome Sciences, University of Maryland School of Medicine, Baltimore, Maryland, USA; ^7^Department of Microbial Pathogenesis, Dental School, University of Maryland, Baltimore, Maryland, USA

*Present address: Department of Laboratory Medicine, University of Washington Medical Center, Seattle, Washington, USA.

This document provides a more detailed description of the bioinformatics analyses presented in the Materials and Methods section entitled ***In vivo*** **Tn-seq analyses**, with the scripts, annotation collection, genome alignments and library metrics used.

**Scripts used**

All preprocessing tasks were performed on the UMIACS computing cluster with scripts produced by CYOA: http://github.com/abelew/CYOA.

Italicized directory names (ending in /) refer to directories within the git repository for this project, italicized scripts are provided in the *scripts/* directory, and italicized data files reside in the *data/* directory.

**Annotation Collection**

GenBank genomes including annotation information were downloaded for the relevant strains. CP008776 is the reference for GAS 5448 genome; CP000017 is the reference MGAS5005 genome. For each of the genbank annotations collected, the genbank file was split into the following: a nucleotide fasta file containing a single genomic sequence, a nucleotide multi-fasta of all annotated coding sequences, a peptide multi-fasta of annotated coding sequences, a gff file of all annotations, a gff file of only the coding sequence annotations, and a gff file describing the inter-cds regions. These may be found in *reference/*. The resulting fasta and gff files were used as the reference for most of the operations performed in this study. Gene ontology information was downloaded from microbesonline.org for strain MGAS5005.

**Genome Alignments**

The approximately 226 million reads of generated from the 5448 *Krmit* Tn-seq libraries were queried for quality with FASTQC (1) before demultiplexing. The *mariner* ITR leading sequences were removed with a combination of Biopieces (https://github.com/maasha/biopieces), Cutadapt (2) and Trimmomatic (3). Reads lacking the ITR sequence were discarded. This process left libraries of *ca*. 200,000,000 reads of lengths primarily 12<=x<=15 nucleotides (**Table SM1**).

These libraries were aligned against the GAS 5448 genome with Bowtie (4, 5) using options to limit the seed sequence to 10 nucleotides (-l 10), allow no mismatch (-v 0), and randomly assign multi-matched reads to one of the possible matching positions (-M 1) (**Fig. SM1**); these are summarized in **Table SM1.** The resulting alignments were converted to sorted/compressed binary alignments (6) and counted (7, 8) against the GAS 5448 genome CDS and intergenic regions.

The resulting count tables are in the *bowtie_mgas_5448/* output directories within *preprocessing/*; and referenced in *sample_sheets/all_samples.xlsx*, which also contains all of the experimental metadata for these experiments and all operations in R. When R functions are noted, they are italicized and have parentheses and may be found in the R markdown transcripts and the hpgltools package: http://github.com/abelew/hpgltools. Annotation data is processed in *annotation.*Rmd, sample metrics may be found in *sample_metrics.*Rmd, and the differential expression operations are in *thytnseq.Rmd* and *subcu.Rmd.* The resulting transcripts are recorded in the associated html/pdf files.

**Library Metrics**

Before using the libraries to determine the fitness for each gene within the 5448 genome, it was necessary to compare and contrast the libraries and quantify their relative coverage, similarity and saturation with respect to available TA insertion points. These comparisons in turn required some attention to the normalization strategies employed.

***Metrics before normalization.***

For the purpose of this study, we primarily focused on the reads containing the ITR sequence, the dinucleotide TA, aligning with 0 mismatches, and mapping within the coding regions (CDS) found on the GAS 5448 chromosome. **Fig. SM2** presents a summary of this analysis for each Tn-seq library.

Additional analyses, i.e. sample densities (**Fig. SM3A**) and box plots (**Fig. SM3B**), provided information regarding the distribution of the numbers of reads per CDS, comparing one Tn-seq read sample to one another. These revealed that the distributions of the numbers of reads per CDS were not necessary consistent across all samples. Moreover, samples with lower numbers of reads do not necessary appear to be all of the lower density samples.

***Metrics after normalization and sample removal.***

As shown previously (**Fig. SM3**), latent variables or unwanted heterogeneity were identified in the Tn-seq read libraries. Datasets were normalized using a log_2_(quantile(cpm(low-filter(counts)))). Further analyses were conducted using the sva package (9) to identify batch effects and additional sources of variation; and an adjustment with the fsva was applied improving clustering of the samples. In these conditions, the sample “mouset48r2v1” was identified as an outliner and was removed from the analysis (**Fig. SM4**).

**Pairwise comparison across time points**

The most expedient method of comparing the libraries was to treat them as if they were components of an RNA sequencing experiment and assuming similar normalization strategies apply (10). This strategy is very similar to that taken by the essentials software package (11), but uses DESeq2 (7, 12) and EdgeR (13, 14). Therefore the alignments were counted by coding sequence and treated as an RNA-seq experiment. The surrogate variable estimates from fsva were added to the models given to DESeq2 (7, 12) and EdgeR (13, 14).

**References for the Supplemental Method section**

1. **Andrews S.** 2010. FASTQC. A quality control tool for high throughput sequence data, *on* Babraham Institute. <http://www.bioinformatics.babraham.ac.uk/projects/fastqc/>.

2. **Martin M.** 2011. Cutadapt removes adapter sequences from high-throughput sequencing reads. EMBnet Journal.

3. **Bolger AM, Lohse M, Usadel B.** 2014. Trimmomatic: a flexible trimmer for Illumina sequence data. Bioinformatics **30:**2114-2120.

4. **Langmead B.** 2010. Aligning short sequencing reads with Bowtie. Current Protocols in Bioinformatics **11**.

5. **Langmead B, Trapnell C, Pop M, Salzberg SL.** 2009. Ultrafast and memory-efficient alignment of short DNA sequences to the human genome. Genome Biology **10:**R25.

6. **Li H, Handsaker B, Wysoker A, Fennell T, Ruan J, Homer N, Marth G, Abecasis G, Durbin R, Subgroup GPDP.** 2009. The Sequence Alignment/Map format and SAMtools. Bioinformatics **25:**2078-2079.

7. **Anders S, Huber W.** 2010. Differential expression analysis for sequence count data. Genome Biology **11:**R106.

8. **Anders S, Pyl PT, Huber W.** 2015. HTSeq--a Python framework to work with high-throughput sequencing data. Bioinformatics **31:**166-169.

9. **Leek JT, Johnson WE, Parker HS, Jaffe AE, Storey JD.** 2012. The sva package for removing batch effects and other unwanted variation in high-throughput experiments. Bioinformatics **28:**882-883.

10. **Dillies MA, Rau A, Aubert J, Hennequet-Antier C, Jeanmougin M, Servant N, Keime C, Marot G, Castel D, Estelle J, Guernec G, Jagla B, Jouneau L, Laloë D, Le Gall C, Schaëffer B, Le Crom S, Guedj M, Jaffrézic F, Consortium. FS.** 2013. A comprehensive evaluation of normalization methods for Illumina high-throughput RNA sequencing data analysis. Briefings in Bioinformatics **14:**671-683.

11. **Zomer A, Burghout P, Bootsma HJ, Hermans PW, van Hijum SA.** 2012. ESSENTIALS: software for rapid analysis of high throughput transposon insertion sequencing data. PLoS One **7:**e43012.

12. **Love MI, Huber W, Anders S.** 2014. Moderated estimation of fold change and dispersion for RNA-Seq data with DESeq2. bioRxiv doi:doi:10.1101/002832.

13. **McCarthy DJ, Chen Y, Smyth GK.** 2012. Differential expression analysis of multifactor RNA-Seq experiments with respect to biological variation. Nucleic Acids Res **40:**4288-4297.

14. **Robinson MD, McCarthy DJ, Smyth GK.** 2010. edgeR: a Bioconductor package for differential expression analysis of digital gene expression data. Bioinformatics **26:**139-140.
