# Supplementary material for: Genome-wide discovery of novel M1T1 group A streptococcal determinants important for fitness and virulence during soft-tissue infection
Source: PLoS Pathog. 2017 Aug 23;13(8):e1006584. doi: 10.1371/journal.ppat.1006584 (PMC5584981; doi:10.1371/journal.ppat.1006584)
Supplement: S4 Table — (PDF) [file ppat.1006584.s010.pdf]

S4 Table. Bacterial strains and plasmids.

Strains and plasmids	Description	Reference our source
***E. coli* strains**		
DH5α	*hsdR17 recA1 gyrA endA1 relA1*	Hanahan and Meselson, 1983
***S. pyogenes* strains**		
5448	M1T1	Chatellier *et al*., 2000
5448ii01780	5448 with insertional inactivation of RS01780 (*pmi*) using pII1538, SpR	This study
5448ii06460	5448 with insertional inactivation of RS06460 (*sagH*) using pII0569, SpR	This study
5448ii02780	5448 with insertional inactivation of RS02780 (*yvqE*) using pII1333, SpR	This study
5448ii08695	5448 with insertional inactivation of RS08695 (*ptsG*) using pII1784, SpR	This study
5448ii04625	5448 with insertional inactivation of RS04625 (*pstS*) using pII0955, SpR	This study
5448ii05865	5448 with insertional inactivation of RS05825 (*vfr*) using pII0693, SpR	This study
5448ii02065	5448 with insertional inactivation of RS02065 (*manL*) using pII1479, SpR	This study
5448ii02090	5448 with insertional inactivation of RS02090 (*cpsA*) using pII1474, SpR	This study
5448ii04065	5448 with insertional inactivation of RS04065 (*dltA*) using pII1073, SpR	This study
5448ii06590	5448 with insertional inactivation of RS06590 (*adcA*) using pII0543, SpR	This study
5448ii00535	5448 with insertional inactivation of RS00535 (*adcR*) using pII0077, SpR	This study
5448ii02880	5448 with insertional inactivation of RS02880 (*rocA*) using pII1318, SpR	This study
5448ii04605	5448 with insertional inactivation of RS04605 (*spxA*) using pII0959, SpR	This study
5448ii08425	5448 with insertional inactivation of RS08425 (*ihk*) using pII1724, SpR	This study
5448KM07790	5448 with *Krmit* transposon insertion in RS07790 (*covS*), KmR	This study
5448KM08410	5448 with *Krmit* transposon insertion in RS08410 (*mga*), KmR	This study
5448KM09015	5448 with *Krmit* transposon insertion in RS09015, KmR	This study
5448KM09010	5448 with *Krmit* transposon insertion in RS09010, KmR	This study
5448KM06895	5448 with *Krmit* transposon insertion in RS06895 (*scfB*), KmR	This study
5448Δ*scfA*	5448 with allelic replacement of RS06890 (*scfA*) by *aphA3*, KmR	This study
5448Δ*scfB*	5448 with allelic replacement of RS06895 (*scfB*) by *aphA3*, KmR	This study
5448Δ*scfAB*	5448 with allelic replacement of RS06890-RS06895 (*scfAB*) by *aphA3*, KmR	This study
**Plasmids**		
pSinS	Suicide plasmid for stable insertional inactivation; Sp^R	Le Breton *et al*., 2015

S4 Table. Bacterial strains and plasmids (continued)

Strains and plasmids	Description	Reference our source
**Plasmids**		
pHlpK	Temperature-sensitive conditional helper vector; Km^R	Le Breton *et al*., 2015
pCRS	Temperature-sensitive conditional vector; Sp^R	Le Breton *et al*. 2013
pII1538	pSinS-based suicide plasmid for insertional inactivation of RS01780; SpR	This study
pII0569	pSinS-based suicide plasmid for insertional inactivation of RS06460; SpR	This study
pII1333	pSinS-based suicide plasmid for insertional inactivation of RS02780; SpR	This study
pII1784	pSinS-based suicide plasmid for insertional inactivation of RS08695; SpR	This study
pII0955	pSinS-based suicide plasmid for insertional inactivation of RS04625; SpR	This study
pII0693	pSinS-based suicide plasmid for insertional inactivation of RS05865; SpR	This study
pII1479	pSinS-based suicide plasmid for insertional inactivation of RS02065; SpR	This study
pII1474	pSinS-based suicide plasmid for insertional inactivation of RS02090; SpR	This study
pII1073	pSinS-based suicide plasmid for insertional inactivation of RS04065; SpR	This study
pII0543	pSinS-based suicide plasmid for insertional inactivation of RS06590; SpR	This study
pII0077	pSinS-based suicide plasmid for insertional inactivation of RS00535; SpR	This study
pII1318	pSinS-based suicide plasmid for insertional inactivation of RS02880; SpR	This study
pII0959	pSinS-based suicide plasmid for insertional inactivation of RS04605; SpR	This study
pII1727	pSinS-based suicide plasmid for insertional inactivation of RS08425; SpR	This study
pAX0478K	pCRS-based plasmid for allelic exchange of the *scfA* ORF; SpR, KmR	This study
pAX0477K	pCRS-based plasmid for allelic exchange of the *scfB* ORF; SpR, KmR	This study
pAX0478-77K	pCRS-based plasmid for allelic exchange of the *scfAB* locus; SpR, KmR	This study
