# Supplementary material for: Genome-wide discovery of novel M1T1 group A streptococcal determinants important for fitness and virulence during soft-tissue infection
Source: PLoS Pathog. 2017 Aug 23;13(8):e1006584. doi: 10.1371/journal.ppat.1006584 (PMC5584981; doi:10.1371/journal.ppat.1006584)
Supplement: S5 Table — (PDF) [file ppat.1006584.s011.pdf]

Table S5. Primers used in this study.

Name and purpose	Sequence (5'- -3') (with underlined feature)	Purpose/target	Feature
**Production of pSinS-based vectors for gene insertional inactivation**			
oll1538F	cccGGATCCATGCACGACAGGATTTGGGG	internal fragment of RS01780 gene (*pmi*)	*BamHI*
oll1538R	cccGGATCCTCTCCTTCATGTTCACGTCC	internal fragment of RS01780 gene (*pmi*)	*BamHI*
M13F	GTTTTCCCAGTCACGACGTTGTA	DNA sequencing of plasmid insert	
M13R	CAGGAAACAGCTATGACC	DNA sequencing of plasmid insert	
oll1538V1	TACGGTTATATCACAACCACTCC	RS01780 mutant genotyping	
oll1538V2	AATAAAATCAATCATCGTAGTCACC	RS01780 mutant genotyping	
oll0569F	cccGGATCCCAGATTTTTAGAAACCGAACAGC	internal fragment of RS06460 gene (*sagH*)	*BamHI*
oll0569R	cccGGATCCAAAACAGCCGTTAAGGCTCC	internal fragment of RS06460 gene (sagH)	*BamHI*
oll0569V1	CCGGTAAAATTACTGTTTTAGG	RS06460 mutant genotyping	
oll0569V2	CGTCACCTGCTTTCTCATACC	RS06460 mutant genotyping	
oll1333F	cccGGATCCGGCTCTACTCAACCATTACC	internal fragment of RS02780 gene (*yvqE*)	*BamHI*
oll1333R	cccGGATCCCTTTTGCATGTTAGCAGTCAGG	internal fragment of RS02780 gene (*yvqE*)	*BamHI*
oll1333V1	AACTACTCGTCATATAAAGATGC	RS02780 mutant genotyping	
oll1333V2	AAATCAACCCCTTCACGTCC	RS02780 mutant genotyping	
oll1784F	cccGGATCCGTCTTCTAACTGCTATTGGTGG	internal fragment of RS08695 gene (*ptsG*)	*BamHI*
oll1784R	cccGGATCCGTGAAAATCTCAGCCAACATGG	internal fragment of RS08695 gene (*ptsG*)	*BamHI*
oll1784V1	ATCGGTTATTTGGTCAATGGTAACC	RS08695 mutant genotyping	
oll1784V2	ATATTTGAAAGAGCAATCATTGG	RS08695 mutant genotyping	
oll0955F	cccGGATCCATTCTTTTTGTTAAGTCTTTTGGC	internal fragment of RS04625 gene (*pstS*)	*BamHI*
oll0955R	cccGGATCCACTGCAAGTCCTGCAACAGC	internal fragment of RS04625 gene (*pstS*)	*BamHI*
oll0955V1	AAAATGAAGATGTTGTGCAGTGG	RS04625 mutant genotyping	
oll0955V2	AGAGCACCAAGATAAGGAATGC	RS04625 mutant genotyping	
oll0693F	cccGGATCCCTAAAGGAAAGTCATTGTTATGC	internal fragment of RS05865 gene (*vfr*)	*BamHI*
oll0693R	cccGGATCCGAACACTAAAACTCAAGTCAACG	internal fragment of RS05865 gene (*vfr*)	*BamHI*
oll0693V1	ACAAGTCTTAAATACCCATAACG	RS05865 mutant genotyping	
oll0693V2	TAGTATCATTAAATTTAATCATCTGC	RS05865 mutant genotyping	
oll1479F	cccGGATCCGCCATGGTAAATTTGCTGAAGG	internal fragment of RS02065 gene (*manL*	*BamHI*
oll1479R	cccGGATCCGTTCAGTGTAAGCTTGAATAAGC	internal fragment of RS02065 gene (*manL*	*BamHI*
oll1479V1	TTAGAGATTACCAAAATCACTCG	RS02065 mutant genotyping	
oll1479V2	AACGACCAAAATTGCAGAAATAATCG	RS02065 mutant genotyping	

Table S5. Primers used in this study (continued).

Name and purpose	Sequence (5'- -3') (with underlined feature)	Role	Feature
**Production of pSinS-based vectors for gene insertional inactivation**			
oII1474F	cccGGATCCCTATTGTGTTAACAACTGTCTTGG	internal fragment of RS02090 gene (*cpsA*)	*BamHI*
oII1474R	cccGGATCCGATAACGTGGTTAAGGTATCTCG	internal fragment of RS02090 gene (*cpsA*)	*BamHI*
oII1474V	TTTACTGTCTGAGACTTAGTTGACC	RS02090 mutant genotyping	
oII1073F	cccGGATCCCAGTTTGCTCAGACACAGGC	internal fragment of RS04065 gene (*dltA*)	*BamHI*
oII1073R	cccGGATCCGTAAGAGGGAATTCCTCAATAGC	internal fragment of RS04065 gene (*dltA*)	*BamHI*
oII1073V	AAGATAACTCCTTACCATCTTC	RS04065 mutant genotyping	
oII0543F	cccGGATCCGCTTGGCAACTTACTCAGGC	internal fragment of RS06590 gene (*acdA*)	*BamHI*
oII0543R	cccGGATCCCATGTCCAGCTCCTGCTACC	internal fragment of RS06590 gene (*acdA*)	*BamHI*
oII0543V	GACGATCTTTGACTTCTTTGTC	RS06590 mutant genotyping	
oII0077F	cccGGATCCGTAAATACTATTTTATTAAAAGC	internal fragment of RS00535 gene (*acdR*)	*BamHI*
oII0077R	cccGGATCCAAGTCACCCTAGCATCAACC	internal fragment of RS00535 gene (*acdR*)	*BamHI*
oII0077V	GGGTAATCATCAGAACGGATTTCC	RS00535 mutant genotyping	
oII1318F	cccGGATCCGTAAGTGCAATTCCGCTTCG	internal fragment of RS02880 gene (*rocA*)	*BamHI*
oII1318R	cccGGATCCGTATTATGTTTAACAACTGAATAAGG	internal fragment of RS02880 gene (*rocA*)	*BamHI*
oII1318V	CTATAGCATTATCTAACAAAAATGAC	RS02880 mutant genotyping	
oII0959F	cccGGATCCGTCGTAAAGCAAGGGCATGG	internal fragment of RS04605 gene (*spxA*)	*BamHI*
oII0959R	cccGGATCCTCCATGATAATAGGGCGACG	internal fragment of RS04605 gene (*spxA*)	*BamHI*
oII0959V	ATCTGCCATCACATCATAAATAAGC	RS04065 mutant genotyping	
oII1724F	5'-cccGGATCCGGTTACCTCTATTTTGTTAGTGC-3'	internal fragment of RS08425 gene (*ihk*)	*BamHI*
oII1724R	5'-cccGGATCCACATTAGAATAAGAGTTGTCAAAGG-3'	internal fragment of RS08425 gene (*ihk*)	*BamHI*
oII1724V	5'-ACATGCTAAGGATACGCCGG-3'	RS08425 mutant genotyping	
**Production of *Krmit* insertion tags**			
Adapter501A	TTCCCTACACGACGCTCTTCCGATCTTATAGCCTNN	*Mme*I Tnseq adapter	Adapter501
Adapter501B	AGGCTATAAGATCGGAAGAGCGTCGTGTAGGGAAAGAG	*Mme*I Tnseq adapter	Adapter501
Adapter502A	TTCCCTACACGACGCTCTTCCGATCTATAGAGGCNN	*Mme*I Tnseq adapter	Adapter502
Adapter502B	GCCTCTATAGATCGGAAGAGCGTCGTGTAGGGAAAGAG	*Mme*I Tnseq adapter	Adapter502
Adapter503A	TTCCCTACACGACGCTCTTCCGATCTCCTATCCTNN	*Mme*I Tnseq adapter	Adapter503
Adapter503B	AGGATAGGAGATCGGAAGAGCGTCGTGTAGGGAAAGAG	*Mme*I Tnseq adapter	Adapter503
Adapter504A	TTCCCTACACGACGCTCTTCCGATCTGGCTCTGANN	*Mme*I Tnseq adapter	Adapter504
Adapter504B	TCAGAGCCAGATCGGAAGAGCGTCGTGTAGGGAAAGAG	*Mme*I Tnseq adapter	Adapter504

Table S5. Primers used in this study (continued).

Name and purpose	Sequence (5'- -3') (with underlined feature)	Role	Feature
**Production of *Krmit* insertion tags**			
Adapter505A	TTCCCTACACGACGCTCTTCCGATCTAGGCGAAGNN	*Mme*I Tnseq adapter	Adapter505
Adapter505B	CTTCGCCTAGATCGGAAGAGCGTCGTGTAGGGAAAGAG	*Mme*I Tnseq adapter	Adapter505
Adapter506A	TTCCCTACACGACGCTCTTCCGATCTTAATCTTANN	*Mme*I Tnseq adapter	Adapter506
Adapter506B	TAAGATTAAGATCGGAAGAGCGTCGTGTAGGGAAAGAG	*Mme*I Tnseq adapter	Adapter506
Adapter507A	TTCCCTACACGACGCTCTTCCGATCTCAGGACGTNN	*Mme*I Tnseq adapter	Adapter507
Adapter507B	ACGTCCTGAGATCGGAAGAGCGTCGTGTAGGGAAAGAG	*Mme*I Tnseq adapter	Adapter507
Adapter508A	TTCCCTACACGACGCTCTTCCGATCTGTACTGACNN	*Mme*I Tnseq adapter	Adapter508
Adapter508B	GTCAGTACAGATCGGAAGAGCGTCGTGTAGGGAAAGAG	*Mme*I Tnseq adapter	Adapter508
Adapter701A	TTCCCTACACGACGCTCTTCCGATCTATTACTCGNN	*Mme*I Tnseq adapter	Adapter701
Adapter701B	CGAGTAATAGATCGGAAGAGCGTCGTGTAGGGAAAGAG	*Mme*I Tnseq adapter	Adapter701
Adapter702A	TTCCCTACACGACGCTCTTCCGATCTTCCGGAGANN	*Mme*I Tnseq adapter	Adapter702
Adapter702B	TCTCCGGAAGATCGGAAGAGCGTCGTGTAGGGAAAGAG	*Mme*I Tnseq adapter	Adapter702
Adapter703A	TTCCCTACACGACGCTCTTCCGATCTCGCTCATTNN	*Mme*I Tnseq adapter	Adapter703
Adapter703B	AATGAGCGAGATCGGAAGAGCGTCGTGTAGGGAAAGAG	*Mme*I Tnseq adapter	Adapter703
Adapter704A	TTCCCTACACGACGCTCTTCCGATCTGAGATTCCNN	*Mme*I Tnseq adapter	Adapter704
Adapter704B	GGAATCTCAGATCGGAAGAGCGTCGTGTAGGGAAAGAG	*Mme*I Tnseq adapter	Adapter703
oKrmit-Tnseq2	CAAGCAGAAGACGGCATACGAAGCGCCTACGAGGAATTTGTATCG	PCR of *Krmit* insertion tags	
oAdapterPCR	AATGATACGGCGACCACCGAGATCACACTCTTTCCCTACACGACGCTCTTCC	PCR of *Krmit* insertion tags	
**Production of pCRS-based vectors for allelic exchange in the *scfAB* locus**			
oKmF	ATGGCTAAAATGAGAATATCACC	promoterless *aphA3* ORF	
oKmR	CTAAAACAATTCATCCAGTAAAATA	promoterless *aphA3* ORF	
oAX0478.1	cccGGATCCCGTAACTGATCTGTCAGTTTTCC	DNA sequence uptream of *scfA*	*BamHI*
oAX0478.2	**GGTGATATTCTCATTTTAGCCAT**TCACCATATCCTTTCTAGATGATAAAAG	DNA sequence uptream of *scfA*	*aphA3*-tail
oAX0478.3	**TATTTTACTGGATGAATTGTTTTAG**ATATATGTCCCAACTCGTGTGTTG	DNA sequence downtream of *scfA*	*aphA3*-tail
oAX0478.4	cccGGATCCATTTTCAGTGGTAATGGTGACC	DNA sequence downtream of *scfA*	*BamHI*
oAX0478.V1	TAATGGGAACAAGCCTGCTTCACG	*PscfA-aphA3* junction genotyping	
oAX0478.V2	GTGAATAATGCCGAAGCGG	*PscfA-aphA3* junction genotyping	
oKmV1	TAGCAGGAGACATTCCTTCC	*PscfA-aphA3* junction genotyping	
oKmV2	TCGAGCTATTTTTTGACTTACTGG	*scfA downstream* junction genotyping	
oAX0477.1	cccGGATCCGGTTCAAAAATACCTACCTAAGC	DNA sequence uptream of *scfB*	*BamHI*

Table S5. Primers used in this study (continued).

Name and purpose	Sequence (5'- -3') (with underlined feature)	Role	Feature
**Production of pCRS-based vectors for allelic exchange in the *scfAB* locus**			
oAX0477.2	**GGTGATATTCTCATTTTAGCCAT**ATAGCCAGCTAAAATTAAAAAGCG	DNA sequence uptream of *scfB*	*aphA3*-tail
oAX0477.3	**TATTTTACTGGATGAATTGTTTTAG**AGACTAATAAAAAAGCACTTGTTTTTTATC	DNA sequence downtream of *scfB*	*aphA3*-tail
oAX0477.4	cccGGATCCGATATACTTGCTGCGCATGC	DNA sequence downtream of *scfB*	*BamHI*
oAX0477.V1	CAATGGTTTGCGATTTTTATGTCC	*scfB-aphA3* junction genotyping	
oAX0477.V2	CTAAAAATGGATATTGATTTCATTACTTCAAAAGGC	*scfB downstream* junction genotyping	
**Verification of the expression of *scfA* and *scfB* in the Δ*scfA* and Δ*scfB* mutants**			
scfA M1T1 RT L	CGCATTCTGTTTGGAACCT		
scfA M1T1 RT R	TTTTTCCAAAAAGCGGTTG		
scfB M1T1 RT L	CAAGGCTGACGAGTCCTTT		
scfB M1T1 RT R	CAGAAACCGTGGTGGAATC		
